# Transformer patient embedding using electronic health records enables patient stratification and progression analysis

**DOI:** 10.1038/s41746-025-01872-z

**Published:** 2025-08-14

**Authors:** Su Xian, Monika E. Grabowska, Iftikhar J. Kullo, Yuan Luo, Jordan W. Smoller, Theresa L. Walunas, Wei-Qi Wei, Gail P. Jarvik, Sean D. Mooney, David R. Crosslin

**Affiliations:** 1 Department of Biomedical Informatics and Medical Education, University of Washington, Seattle, WA USA; 2 Department of Biomedical Informatics, Vanderbilt University Medical Center, Nashville, TN USA; 3 Department of Cardiovascular Medicine and the Gonda Vascular Center, Mayo Clinic Rochester Minnesota, Rochester, MN USA; 4 Department of Preventive Medicine, Northwestern University Feinberg School of Medicine, Chicago, IL USA; 5 Psychiatric and Neurodevelopmental Genetics Unit, Center for Genomic Medicine, Massachusetts General Hospital, Boston, MA USA; 6 Center for Precision Psychiatry, Department of Psychiatry, Massachusetts General Hospital, Boston, MA USA; 7 Department of Medicine, Northwestern University Feinberg School of Medicine, Chicago, IL USA; 8 Department of Medicine, Division of Medical Genetics, University of Washington, Seattle, WA USA; 9 Department of Genome Sciences, University of Washington, Seattle, WA USA; 10 Center for Information Technology, National Institutes of Health, Bethesda, MD USA; 11 Department of Medicine, Division of Biomedical Informatics and Genomics, Tulane University, New Orleans, LA USA

**Keywords:** Gastrointestinal cancer, Tumour heterogeneity, Cancer, Diseases, Cancer, Immunological disorders

## Abstract

Current studies regarding the secondary use of electronic health records (EHR) predominantly rely on domain expertise and existing medical knowledge. A powerful representation approach can unleash the potential of discovering new medical patterns underlying the EHR. Here, we introduce an unsupervised method for embedding high-dimensional EHR data at the patient level to characterize heterogeneity in complex diseases and identify novel disease patterns linked to disparities in clinical outcomes. We applied this approach to 34,851 unique medical codes across 1,046,649 longitudinal patient events, including 102,740 patients in the Electronic Medical Records and GEnomics (eMERGE) Network. The model achieved strong predictive performance in predicting future disease (median AUROC = 0.87 within one year) and bulk phenotyping (median AUROC = 0.84). Notably, these patient embeddings revealed diverse comorbidity profiles and health outcomes, including distinct subtypes and progression patterns in colorectal cancer and systemic lupus erythematosus.

## Introduction

Electronic health records (EHRs) have become widely adopted across the United States^1^. The “meaningful use” of EHR, as defined by the Department of Health and Human Services, aims to improve the quality and efficiency of care^2^. In recent decades, the potential for the secondary use of EHR to facilitate clinical research has been explored and gained considerable attention. One of the major topics is EHR-based digital phenotyping^3,4,5^. The electronic Medical Records and GEnomics (eMERGE) consortium created the Phenotype KnowledgeBase (PheKB) as a digital platform for phenotyping knowledge. This initiative allows multiple large hospitals and universities to collaborate and share their phenotyping algorithms developed from electronic health record (EHR) data^6,7^. Though most of the algorithms stored in PheKB are rule-based and validated by domain expertise, there are also efforts towards developing scalable machine learning algorithms for EHR-based phenotyping, which generally require less time and expertise. Various machine learning and deep learning methods have been applied to build EHR-based phenotyping algorithms, including support vector machines (SVM), random forests, logistic regressions, and neural network architectures^4,8,9^. These efforts seek to enhance our understanding of diseases and improve the healthcare system. Currently, a crucial and challenging question is how to leverage the EHR data to help identify and characterize disease patterns. With the ideal solution, we can promote disease monitoring and clinical predictive tasks.

Representation learning is a powerful tool that can characterize existing and uncover novel disease patterns to facilitate the study of disease etiology, prevention, forecasting, and even heterogeneity analysis^10–13^. Though researchers in modern times are exploring new patterns using the EHR data^14^, the research applications of EHR still largely depend on existing domain knowledge from experts^15–18^. Emerging pioneering studies using EHRs are uncovering the heterogeneity of a defined phenotype, highlighting differences in comorbidities that may be linked to genetics, lifestyle, and environmental factors^19–21^. To further improve this, representation learning has become an essential tool for handling high-dimensional EHR data, aiding in pattern recognition, heterogeneity analysis, and subsequent prediction tasks. However, a significant challenge is that, in the EHR, each patient can have multiple visits in a year or across different years, generating a large number of diagnosis and procedure codes accompanied by numerous lab values and observations, making it difficult to summarize. A simple representation of patients utilizing the nature of the EHR data structure is through binary vector encoding to indicate if patients have specific diagnoses, procedures, or labs. In this context, matrix decomposition serves as an effective tool to compress patient data into lower numerical dimensions.^22^. Matrix decomposition, such as principal components analysis (PCA) and non-negative matrix factorization (NMF), is used to compress a large matrix of patients into a relatively smaller one, while preserving the relationship of the selected features, such as diagnosis, procedures, and labs. The resulting matrix is commonly referred to as patient embedding. Besides traditional matrix decomposition, a specific deep-learning model adopted from the autoencoder has been implemented to perform the embedding task^10^. Apart from the purely data-driven method, some methods integrate medical entities to perform predictive tasks^23^. Overall, without the obligation of cohort building or domain expertise to iteratively process the phenotyping tasks, various unsupervised methods are applied to identify unknown patterns of patients and diseases using the EHR data.

Many neural network architectures have been developed and proven to show progress in various kinds of health-related tasks, both supervised and unsupervised^24,25^. Specifically, transformer attention mechanisms have surpassed traditional sequence models, such as recurrent neural networks (RNN) and their variations (gated recurrent unit, GRU, long short-term memory, LSTM), both in efficiency and performance in downstream tasks within the natural language processing (NLP) field^26,27^. Inspired by the attention mechanisms, we explored the potential of using medical diagnosis and procedures to construct patient vectors. In this work, we represented each patient as a sentence, using diagnosis and procedure codes as vocabularies to compose patient vectors. Moreover, we generated patient vectors in a longitudinal format, allowing investigation of a patient at a specific time point. Here, we present the transformer-based EHR patient data embedding method and demonstrate its power in bulk phenotyping, future disease prediction, comorbidity study, and longitudinal analysis. We utilized 34,851 available codes for 1,046,649 patients from the electronic Medical Records and Genomics (eMERGE) network to train the model and generate patient embeddings. The patient embeddings demonstrated exceptional performance in forecasting future disease events (median ROC = 0.87) and bulk phenotyping (median ROC = 0.84) using simple logistic regression models without any fine-tuning. More importantly, we demonstrated that patient vectors can reveal the heterogeneity of comorbidity patterns within a single phenotype, which forms sub-clusters of comorbidities. We also showed that these clusters exhibit different trajectories of disease progression over time. With external validation using de-identified patients from UW spanning 20 years (*n* = 840,000, from 2000 to 2020), our model showed great flexibility and stable performance in reproducing the sub-cluster analysis and identifying distinct clinical outcomes associated with each cluster. Together, these findings provide new insights into personalized medicine by analyzing complex patterns of comorbidity within diseases and revealing distinct disease trajectories associated with varying mortality rates.

## Results

### Model illustration and performance evaluation

**Fig. 1:**
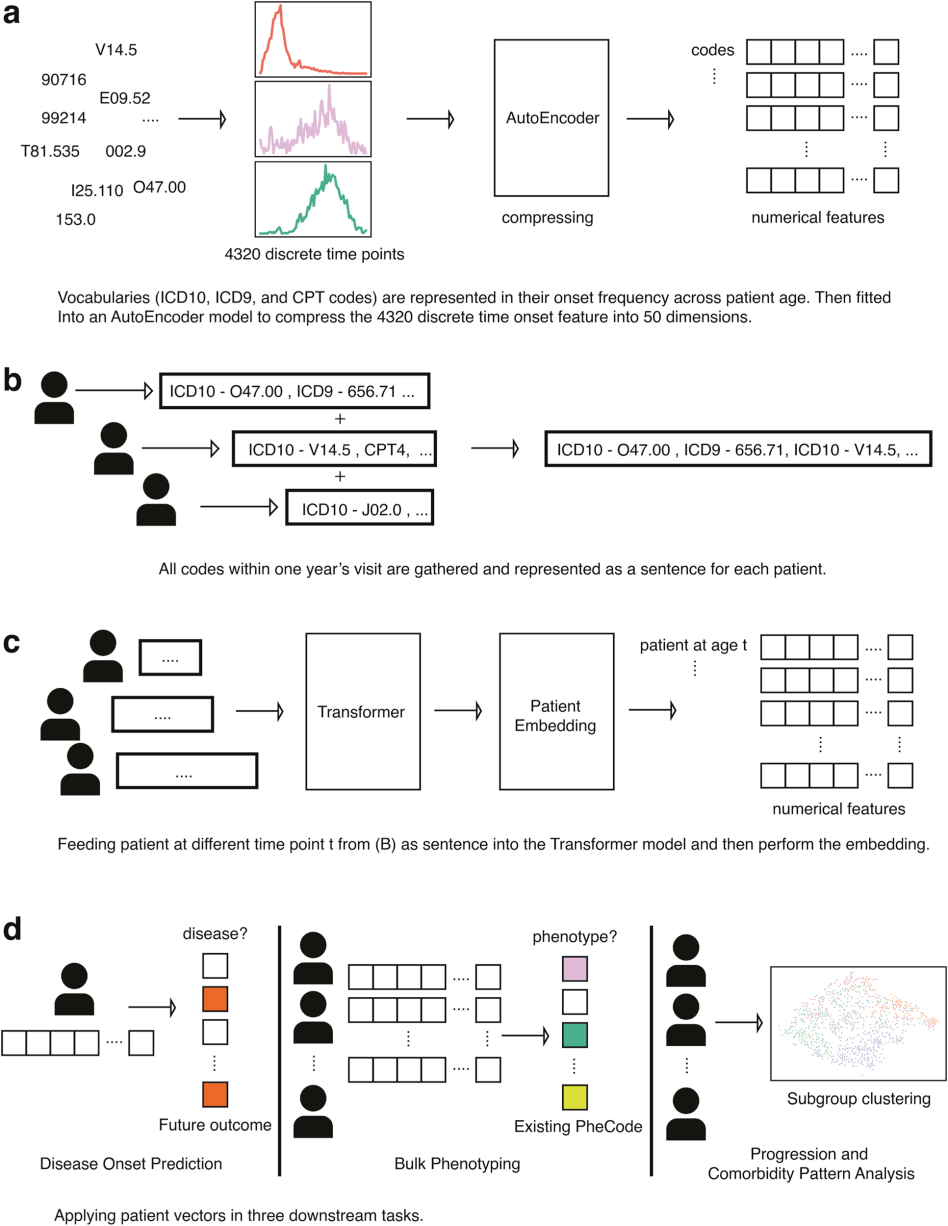
Illustration of the patient embedding model and downstream application. **a** Encoding diagnosis and procedure codes into numerical space as basic vocabularies in downstream training using an autoencoder architecture. **b** Representing patients’ visits within a year as sentences and diagnoses, procedure codes as vocabularies. **c** Feed each visit from **b** into a Transformer model and concatenate through the Patient Embedding model to generate the final patient vectors. **d** Downstream applications workflow of disease onset prediction (left), bulk phenotyping (middle), and clustering subgroup analysis (right).

The outline of our work is summarized in Fig. 1, with a detailed model architecture in Fig. S1. The three steps of performing numerical patient embedding in this session are illustrated in Fig. 1a–c. We first created an embedding of vocabularies (diagnosis codes, procedure codes) using a variational autoencoder neural network architecture (Figs. 1a, S1, left). Then, we fed the embedded diagnosis codes and procedure codes as vocabularies to a transformer model, representing each patient’s longitudinal visits as sentences (Figs. 1b, c, S1, middle and right). Finally, we implemented the sentence-BERT architecture, transforming the 2-D vector output (vocabulary × probability matrix) from the transformer model into a 1-D vector, which serves as the final output of patient embedding (Figs. 1c and S1). We evaluated each step separately with a greater emphasis on the last two steps (Fig. 1b, c), as they are critical for the performance of important downstream tasks. For more detail, we included the distribution of the number of follow-up years and number of codes per year in Figure S2.

During training, the transformer model masked 20% of the code, and we evaluated its ability to use existing codes to predict the codes for the following year. The model achieved a peak prediction accuracy of 79%. The result suggests that the learned numerical embeddings can almost perfectly recapture what is shown in the training set. However, inferring 20% of the masked code using the provided information remains a challenging task. During the evaluation, we randomly selected 32,000 samples (1000 steps with a batch size of 32) without masking the sequence. The model achieved a mean precision of 91.7% and a median precision of 95.8%. Additionally, it attained a mean recall of 89.5% and a median recall of 93.7% in reconstructing the original codes (Table 1). These results demonstrate that the numerically embedded vector can accurately recover the diagnosis and procedure codes.

**Table 1:** Evaluation of sequence recovery performance

	Median	Mean
Precision	0.958 (0.90, 1.00)	0.917 ± 0.096
Recall	0.937 (0.875, 0.964)	0.895 ± 0.092

The sentence-embedding model (Fig. S1, right) has two goals. One is to predict whether a given pair of patient embeddings at different time points represents the same patient. Another goal is to predict if one patient embedding is a longitudinal following event of another. We selected 34,633 events from 500 randomly chosen patients during the evaluation. We formed pairs as input and divided them into 5 iterations (with 100 patients for each iteration), and evaluated the performance of two tasks (Table 2). The accuracy for the *is_same_patient* task is 0.797 ± 0.0016 (mean and standard deviation), and for *is_next_event* is 0.769 ± 0.011. Overall, the model achieved ideal performances on two crucial tasks, indicating a great potential for multi-purpose downstream analysis.

**Table 2:** Evaluation of the *is_same_patient* and the *is_next_event* performance for the sentence-embedding model

	*is_same_patient*	*is_next_event*
Accuracy	0.797 ± 0.0016	0.769 ± 0.011

### Disease onset prediction and bulk-phenotyping

**Fig. 2:**
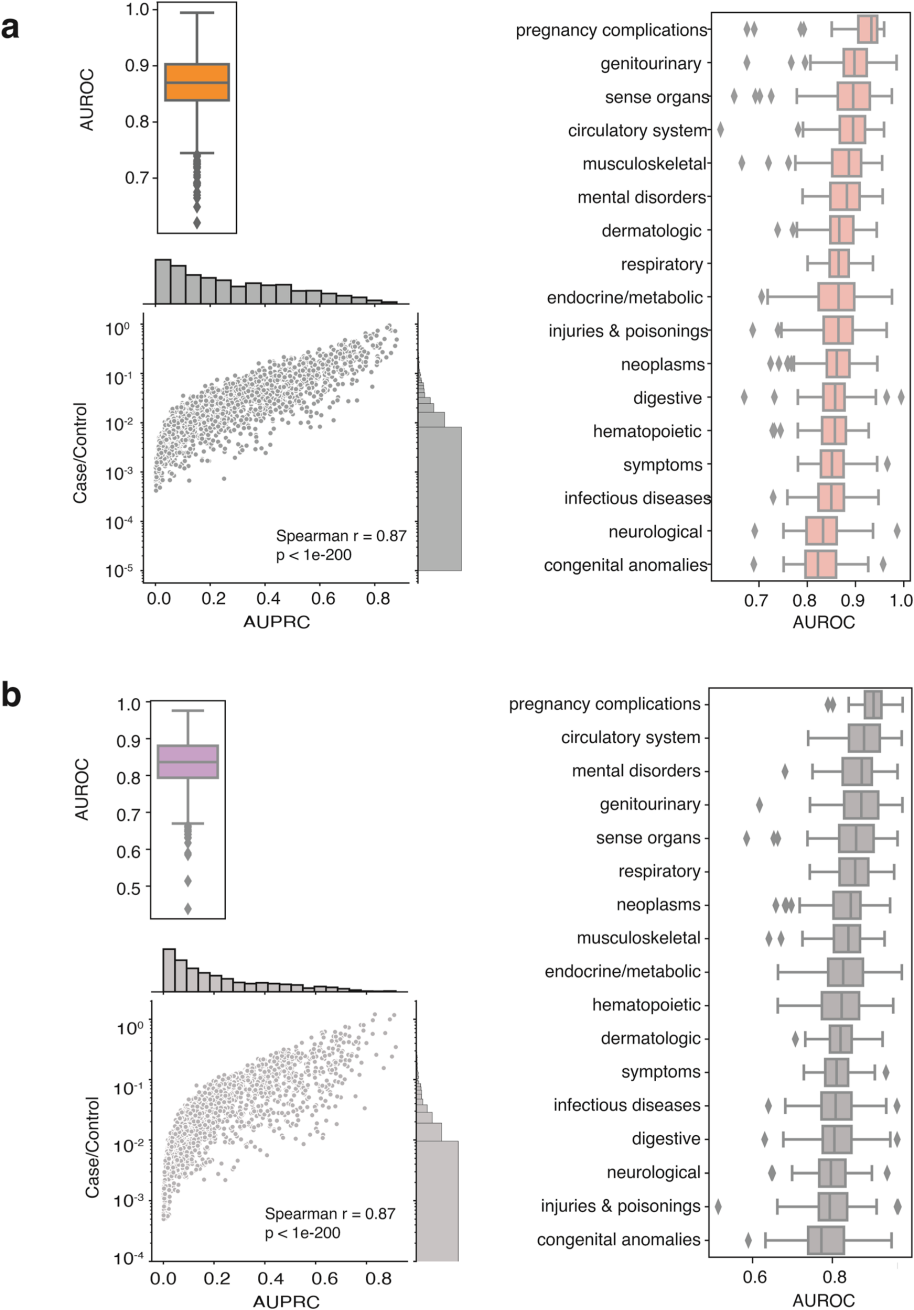
Model performance on (a) disease onset prediction and (b) bulk phenotyping. Performances on disease onset prediction (**a**) and bulk phenotyping (**b**). The top right shows the boxplot of AUROC, and the bottom shows the relationship between sample size (positive/negative ratio) and AUPRC. On the right, each boxplot represents the AUROC distribution categorized by disease class according to phecodes.

Predicting disease onset is vital for early diagnosis and risk assessment before the disease occurs. Using the longitudinal embeddings of patient vectors, we first construct simple logistic regression models to distinguish between the disease state and the non-disease state (Fig. S3a). We mapped ICD-10 and ICD-9 codes to phecodes as a standard reference of phenotypes^28^. For a specific disease represented by phecodes, the patient vector after a diagnosis time point is denoted as positive samples (disease state), and the patient vectors before the diagnosis are negative samples (non-disease state). Across all 1855 unique phenotypes represented by phecodes, the simple logistic regression model achieved a median area under the receiver operating characteristic (AUROC) of 0.81. Among the groups analyzed, the circulatory system group showed the highest performance, while the pregnancy complications group ranked the lowest (Fig. S3b). The median area under the precision–recall curve (AURPC) is 0.80, which is not surprising, as the sample sizes of the positive and negative samples are approximately balanced (Fig. S3b). This disease state versus non-disease state classification task can be seen as a baseline between “phenotyping” and “onset prediction” as it demonstrates the ability to differentiate between disease and non-disease states. However, it lacks timing in prediction. Therefore, we then performed disease onset prediction using patient vectors one year before disease onset (Figs. 2a and S4a). We observed a median AUROC of 0.87 for the disease onset task. The best-performing group was pregnancy complications (AUROC = 0.93), while the lowest-performing group was congenital anomalies (AUROC = 0.82). These results exceeded the performance of the baseline model, which distinguishes between disease status and non-disease status. We attribute this exceeding performance to the fine-tuning tasks from the patient embedding model, where we trained the longitudinal vectors to predict the next event (Figs. 1c and S1, middle). Although the sample size is heavily imbalanced within each phenotype for this task, we show that the AUPRC is strongly associated with the positive-to-negative ratio (Fig. 2a), indicating stable performance when enough samples are available. These results suggest that patient vectors can be used as features for disease forecasting tools or risk models, even when applying simple linear models.

Automated bulk phenotyping is ideal if no extra human effort is required, as developing high-performance phenotyping algorithms is usually iterative, time-consuming, and demands knowledge from domain expertise^15,29^. Here, we explored the potential for using the longitudinal vectors as bulk-phenotyping tools and illustrated the process in Figure S4b, using phecodes as standard references. In detail, since each patient has multiple vectors corresponding to different time points, we calculated the arithmetic mean of those vectors as a compressed vector representation of each patient. This method resembles the chronological accumulation of all high-dimensional vectors and normalizes it by the number of steps (referred to as summed-up embedding later). The median AUROC of all 1855 phenotypes is 0.84, which is slightly lower than the disease onset task (Fig. 2b). The best performance group is for pregnancy complications (AUROC = 0.90), and the poorest for congenital abnormalities (AUROC = 0.77). Likewise, we observed a strong association between the positive-to-negative ratio and the AUPRC (Fig. 2b). Together, these two outstanding performances showed great potential for patient vectors to achieve excellent results in bulk phenotyping.

We benchmarked our model’s performance in predicting disease onset, comparing it to Deep Patient and BEHRT, two models specifically trained for future disease prediction (see Supplementary Table 6 and methods for more details)^30^. We show that the embedding quality of the transformer is on par with the state-of-the-art BEHRT model in disease prediction, albeit with 0.022 lower median AUROC and 0.012 lower mean AUROC. Although we observed a performance drop after fine-tuning with the S-BERT model, we reason that the S-BERT model is fine-tuned on patient-level information and not prioritized for disease onset prediction. We will demonstrate the power of this fine-tuned embedding in the following sub-phenotype session.

### Sub-phenotype identification using patient vectors clustering analysis

**Fig. 3:**
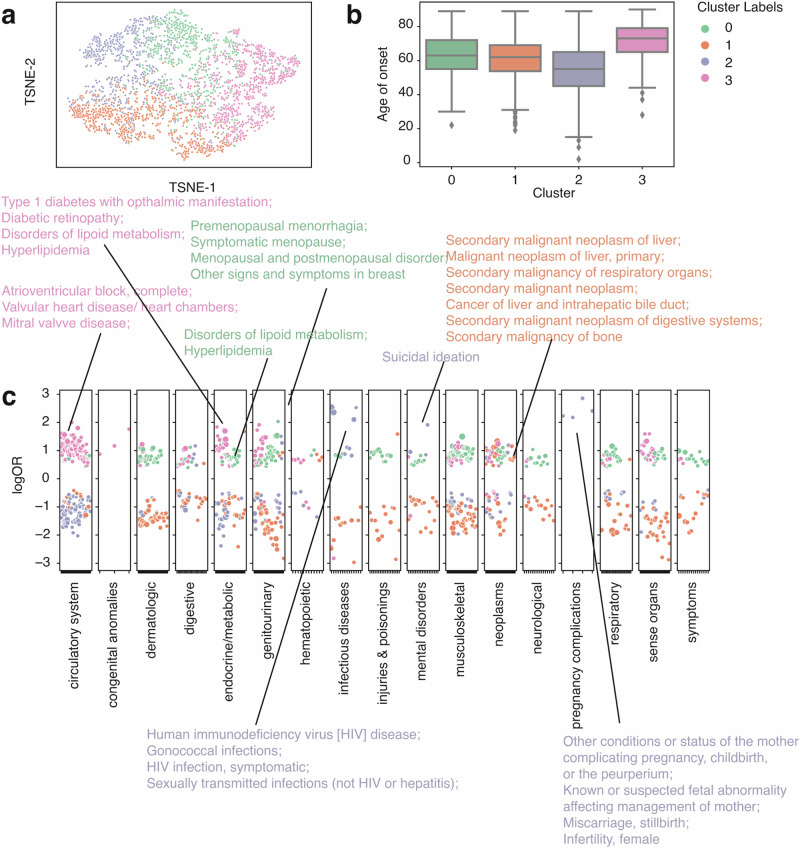
Clustering analysis identified subgroups with distinct comorbidity patterns in colorectal cancer patients (*n* = 2837) from the eMERGE cohort. **a** TSNE plot of patient vectors colored by cluster groups defined using a Gaussian mixture model (GMM) with optimal Bayesian information criteria (BIC). **b** Box plot showing the distribution of age of onset for individual CRC cluster groups. **c** Comorbidity pattern enrichment plot grouped by disease classes (in the *x*-axis) within each cluster group (represented by color). The *y*-axis indicates the log odds ratio of the comorbidity enrichment. Only statistically significant results are shown (*p* < 2e−5) after the Bonferroni correction. Colored texts are used to highlight the top results within each cluster group.

Comorbidity, the simultaneous presence of two or more diseases or medical conditions, has a profound impact on an individual’s care plan, quality of life, and mortality ^31^. Using the summed-up embedding to represent individual patients, we show that within a well-defined single phenotype, the comorbidity status formed different clusters, exhibiting heterogeneity in comorbidity patterns (Fig. 3). Using colorectal cancer (CRC) patients (identified using phecode 153) as an example (*n* = 2837), we identified 4 clusters (Fig. 3a) according to the Bayesian information criterion (BIC) using Gaussian mixture models (GMM) (Fig. S5a). To characterize the comorbidity patterns within each cluster group, we fitted logistic regression models using one cluster group versus the rest strategy adjusted for age of onset, race, ethnicity, and sites (see the “Methods” section). Cluster 2 (median onset age = 51) has the earliest onset age (Fig. 3b) and is strongly associated with HIV infection (phecode = 071) and a few pregnancy complications, representing a subgroup of female patients with immunodeficiency phenotypes (Fig. 3c). This association between HIV and CRC is not new and is more prevalent in women in a pooled result from several studies^32–35^. Cluster 1 (median onset age = 62) is associated with secondary malignant neoplasm, which reflects cancer pleiotropy and late-stage cancer patients with metastasis (Fig. 3c) ^36,37^. Cluster 0 (median onset age = 60) is enriched in genitourinary and endocrine diseases, including disorders of lipoid metabolism, menopause issues, and menorrhagia issues (Fig. 3c). Though endocrine and metabolic disease might be risk factors for CRC, and vice versa, a study has also shown a greater risk of CRC patients developing endocrine and metabolic diseases^38,39^. Cluster 3 (median onset age = 72) is the latest onset group (Fig. 3b), which has a strong pattern of the circulatory system and endocrine diseases, including atrioventricular block, valvular heart disease, mitral valve disease, diabetes, and hyperlipidemia (Fig. 3c). This cluster group aligns with existing findings that CRC patients have an increased risk of developing cardiovascular disease and heart failure^40,41^.

**Fig. 4:**
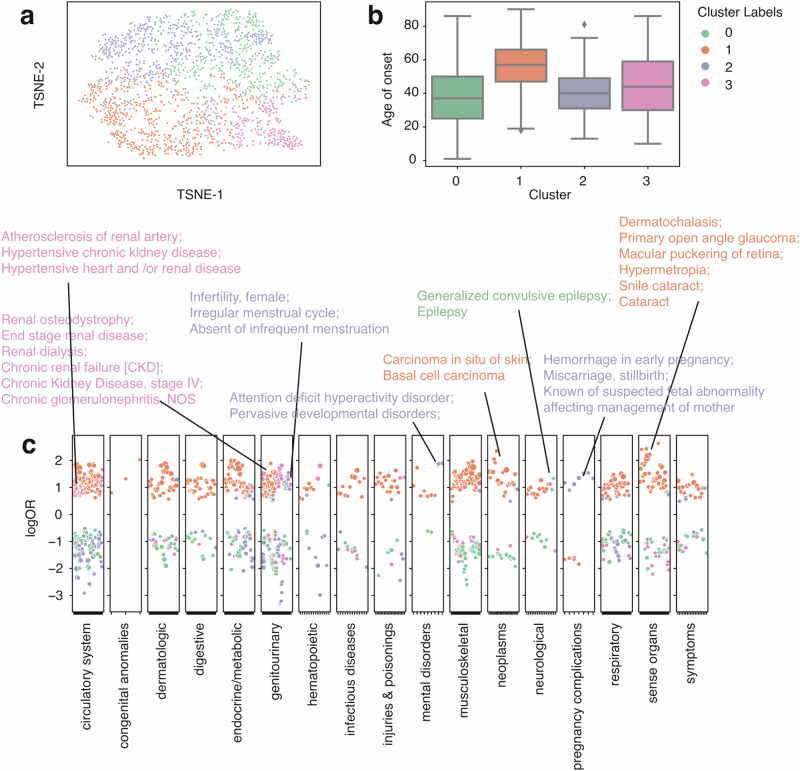
Clustering analysis identified subgroups with distinct comorbidity patterns in systemic lupus erythematosus patients (*n* = 1806) from the eMERGE cohort. **a** TSNE plot of patient vectors colored by cluster groups defined using a Gaussian mixture model (GMM) with optimal Bayesian information criteria (BIC). **b** Box plot showing the distribution of age of onset for individual SLE cluster groups. **c** Comorbidity pattern enrichment plot grouped by disease classes (in the *x*-axis) within each cluster group (represented by color). The *y*-axis indicates the log odds ratio of the comorbidity enrichment. Only statistically significant results are shown (*p* < 2e−5) after the Bonferroni correction. Colored texts are used to highlight the top results within each cluster group.

Similarly, we performed GMM clustering on systemic lupus erythematosus (SLE) patients (*n* = 1806, Fig. 4). We identified 4 clusters (Fig. 4a) based on BIC and observed a wide range of disease patterns within these clusters (Fig. S5b). Cluster 0 has the lowest onset age (median onset age = 37, Fig. 4b) among all other groups and is associated with epilepsy (Fig. 4c). Cluster 1 has the highest median onset age (median onset age = 57, Fig. 4b) and is joint with skin cancer and eye diseases, such as glaucoma, cataracts, dermatochalasis, etc (Fig. 4c). Evidence shows that SLE patients can develop cataracts and many other eye diseases^42,43^, some of which could be linked to medications^43^. Cluster 2 has a median onset age similar to cluster 0 (median onset age = 40, Fig. 4b) and is enriched in pregnancy complications, including hemorrhage in early pregnancy, miscarriage, stillbirth, etc (Fig. 4c). Cluster 2 also has signs of infertility, irregular menstrual cycle, and developmental disorder (Fig. 4c). Though still unclear, numerous studies have tried to dissect the relationship between lupus and pregnancy complications and identified hormone-level abnormalities^44,45^. Cluster 3 has a median onset age of 44 and is associated with renal diseases, including renal osteodystrophy, end-stage renal disease, chronic kidney disease, etc (Fig. 4c). SLE is known to be a systemic disease, and cluster 3 reflects its systemic involvement in kidney disorders. When aggravated, it can lead to kidney failure. Using CRC and SLE as two case studies, we show that patient vectors can reveal distinct comorbidity patterns. Even within a single phenotype, diverse patterns of comorbidities exist. Thus, further evaluation and a personalized care plan are required to improve healthcare.

In addition to the two mentioned phenotypes above, we also present the visualization of other phenotypes that have a reasonable sample size. The interactive website is running on: https://ehrcluster.web.app/. Users can search for a phenotype of interest and visualize the subgroup clusters.

### External evaluation using UW EHR data identified similar comorbidity patterns and survival differences

To the best of our knowledge, most of the unsupervised patient representation learning models are only evaluated internally, without robust external validation. Here, we collected the EHR data at the University of Washington (UW) from 2000 to 2020, including *n* = 840,000 patients as external sources of validation to assess our model’s validity and robustness. First, albeit not comparable to the original model performances (Table 1), the transformer model trained on the eMERGE data still earned a reasonable performance in sequence recovery tasks running on UW patients, with a mean and median precision of 0.862 and 0.903, respectively. The mean and median recall are 0.852 and 0.896 (Table 3). Next, the performance of the sentence embedding model has an accuracy of 0.796 ± 0.0023 in the *is_same_patient* task and 0.742 ± 0.0042 for the *is_next_event* task on the UW dataset. This result is interesting as it is comparable to the performance on the training set (eMERGE), and we can conclude that the performance is ideal for external validation (Table 4). Together, this evidence indicates that the transformer model excels in patient embedding internally and demonstrates generalizability with the external UW cohort.

**Table 3:** External validation of the *is_same_patient* and the *is_next_event* performance for the sentence-embedding model using the UW cohort

	Median	Mean
Precision	0.903 (0.833,0.946)	0.862 ± 0.129
Recall	0.896 (0.813, 0.938)	0.852 ± 0.128

**Table 4:** Evaluation of the *is_same_patient* and the *is_next_event* performance for the sentence-embedding model using the UW cohort

	*is_same_patient*	*is_next_event*
Accuracy	0.796 ± 0.0023	0.742 ± 0.0042

We then evaluated the performance of bulk phenotyping and disease onset prediction on the UW dataset (Fig. S6). The median AUROC for the bulk phenotyping task and disease onset task is 0.83 and 0.84, respectively. We noticed a tiny performance drop in the UW datasets from comparing these two tasks to the original eMERGE dataset. Still, the performance is robust and surprisingly stable for an external evaluation (Fig. S6).

**Fig. 5:**
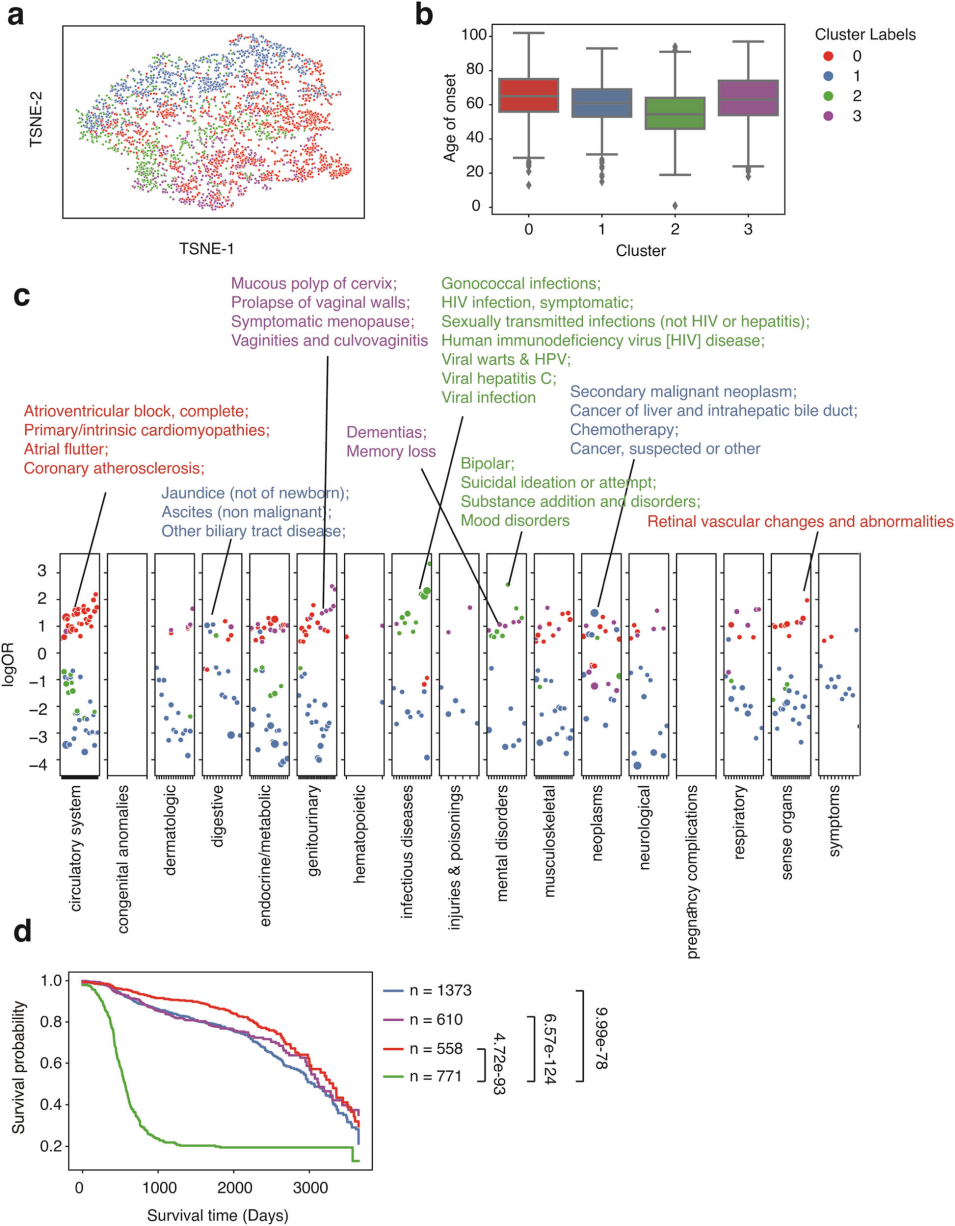
Clustering analysis identified subgroups with distinct comorbidity patterns in colorectal cancer patients (*n* = 3312) from the UW cohort. **a** TSNE plot of patient vectors colored by cluster groups defined using a Gaussian mixture model (GMM) with optimal Bayesian Information Criteria (BIC). **b** Box plot showing the distribution of age of onset for individual CRC cluster groups. **c** Comorbidity pattern enrichment plot grouped by disease classes (in the *x*-axis) within each cluster group (represented by color). The *y*-axis indicates the log odds ratio of the comorbidity enrichment. Only statistically significant results are shown (*p* < 2e−5) after Bonferroni correction. Colored texts are used to highlight the top results within each cluster group. **d** Kaplan–Meier curve showing 10-year overall survival differences across individual cluster groups.

Besides the model performance, bulk-phenotyping, and onset prediction, more importantly, we also performed the comorbidity analysis to see if we could re-identify similar disease patterns across distinct groups. We focused on SLE and CRC and compared the results between the UW cohort and the eMERGE cohort, aiming to reproduce the findings in the UW validation cohort. We discovered 4 clusters of CRC in the UW cohort (Fig. 5a). One cluster (cluster 2, the median age of onset = 54.5) that has a relatively younger age of onset is enriched in infectious diseases (HIV infection, Viral hepatitis C, etc.) and a few mental disorders (Bipolar, Suicidal ideation or attempt, Mood disorder, etc.), identical to cluster 2 from the eMERGE cohort (Fig. 5b, c). Cluster 0 (median age of onset = 65, Fig. 5b, c) from the UW cohort is similar to cluster 3 from the eMERGE cohort (Fig. 3b, c), as both showed phenotype enrichment in circulatory systems (Atrioventricular block) and endocrine/metabolic diseases. Moreover, cluster 1 (median age of onset = 61, Fig. 5b) from the UW and eMERGE cohorts (Fig. 3b, c) are both enriched in secondary malignant neoplasm, specifically, cancer of the liver and intrahepatic bile duct. Together, these results provided compelling evidence that our findings of disease subtypes from the training cohort (the eMERGE cohort) are stable and can be validated using the UW cohort externally. Again, analyzing the 10-year overall survival difference (Fig. 5d), we found cluster 2, enriched in infectious diseases and mental disorders, showed a significantly lower overall survival probability than the other 3 clusters. Searching through existing literature, though there are a few discussions about HIV and colorectal cancer risk, only one report used a meta-analysis method investigating the mortality rate of CRC patients with HIV, with non-significant results, partially due to inadequate samples (*n* = 194)^32^. One report also found HIV-infected cancer patients with elevated mortality rates^46^. Our findings provide supporting evidence supporting these research works and demonstrate the potential of uncovering new patterns in clinical outcomes among patients. Most current research often overlooks the impact of comorbidity patterns on outcome prediction and personalized medicine. Our data indicates that recognizing these patterns can provide essential insights into patient care and life expectancy.

**Fig. 6:**
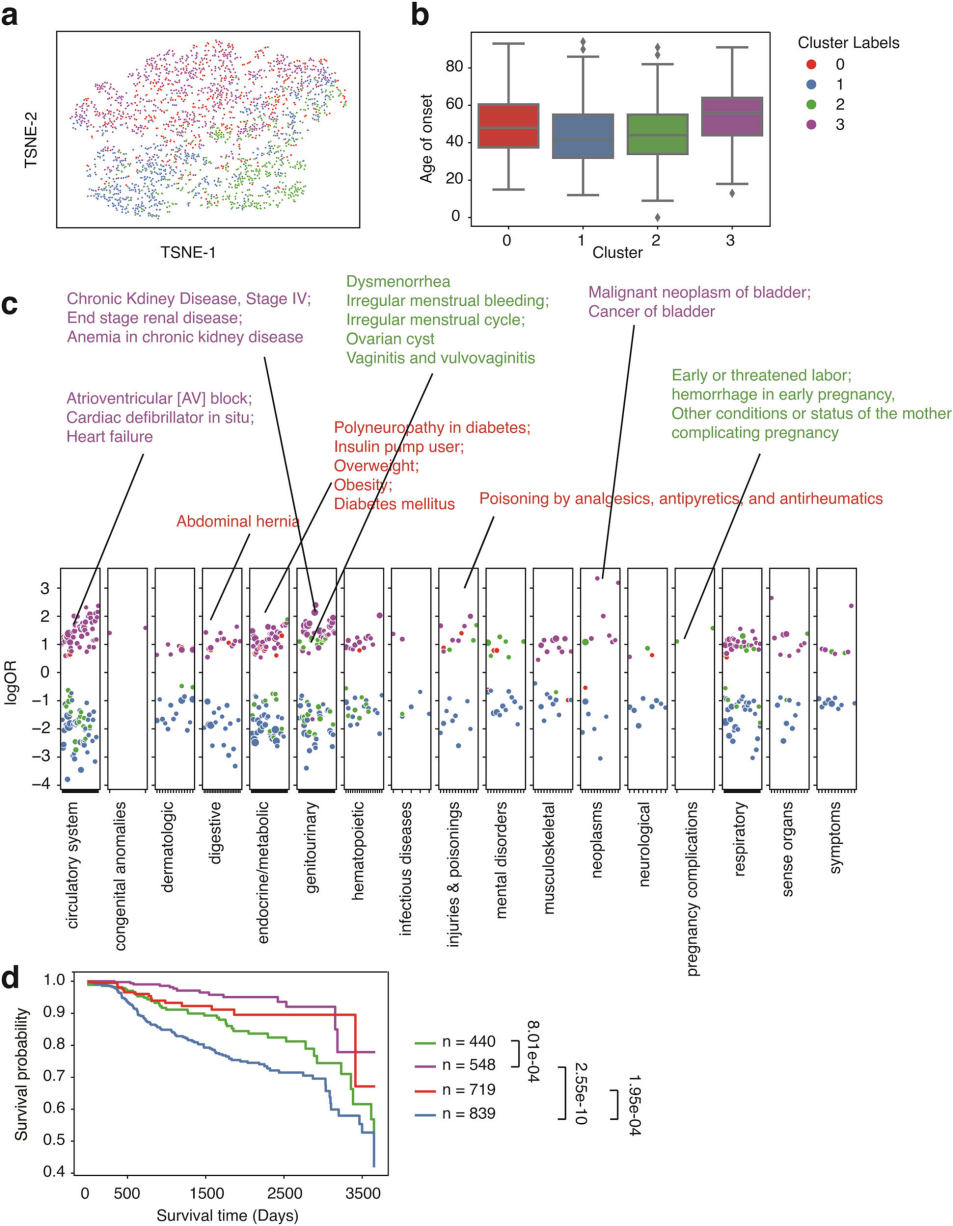
Clustering analysis identified subgroups with distinct comorbidity patterns in systemic lupus erythematosus patients (*n* = 2546) from the UW cohort. **a** TSNE plot of patient vectors colored by cluster groups defined using Gaussian mixture model (GMM) with optimal Bayesian Information Criteria (BIC). **b** Box plot showing the distribution of age of onset for individual SLE cluster groups. **c** Comorbidity pattern enrichment plot grouped by disease classes (in the *x*-axis) within each cluster group (represented by color). The *y*-axis indicates the log odds ratio of the comorbidity enrichment. Only statistically significant results are shown (*p* < 2e−5) after the Bonferroni correction. Colored texts are used to highlight the top results within each cluster group. **d** Kaplan–Meier curve showing 10-year overall survival differences across individual cluster groups.

In the UW cohort, we identified 4 clusters of SLE enriched for different comorbidities (Fig. 6a, b). Cluster 2 (median age of onset = 44), likewise, enriched with pregnancy complications, genitourinary, and a few mental disorders, has a relatively early onset age and is highly similar to what we identified in the eMERGE cohort. Cluster 0 (median age of onset = 48, Fig. 6b) is associated with endocrine/metabolic (such as diabetes, overweight, and obesity, Fig. 6c). Cluster 3 (median age of onset = 56, Fig. 6b) is associated with the circulatory system (Atrioventricular block, Cardiac defibrillator in situ, Heart failure, etc, Fig. 6c), genitourinary (chronic Kidney diseases, end-stage renal disease, anemia in chronic kidney disease, etc, Fig. 6c.), and a few neoplasms (malignant neoplasm of bladder, cancer of bladder). Cluster 1 (median age of onset = 42, Fig. 6b) does not have a unique pattern of enrichment of comorbidities (Fig. 6c). In short, we identified 4 clusters of SLE in the UW cohort, with three having distinct disease patterns. Two cluster groups have identical properties to what we have found in the eMERGE dataset, including pregnancy-complication-associated lupus (cluster 2) and renal-associated lupus (cluster 3). Additionally, with available survival data in the UW cohort, we compared the 10-year overall survival among different cluster groups (Fig. 6d). Among the comorbidity groups, cluster 1 showed no comorbidity enrichment and had the lowest 10-year survival rate. We reason that the low survival rate may be partially attributed to a higher proportion of males in cluster 1 compared to other clusters (odds = 2.54, *p* = 1.42e−14). This is consistent with previous reports that male SLE patients suffer from a lower life expectancy compared to females^47,48^. Then, we noticed that cluster 2 also showed a relatively lower survival rate than Cluster 3 (FDR = 8.01e−4). This result might imply that pregnancy-complication-associated lupus patients might need more follow-up and on-time treatment to improve their health outcomes and life expectancy. One national study also found that SLE women have 20-fold higher maternal death^44^. Clusters 0 (diabetes-associated SLE) and cluster 3 (renal-associated SLE) showed a better survival status, which also has a relatively late onset age, potentially representing late-stage SLE, as SLE can progress into multiple organ-level dysfunctions.

To further illustrate the clustering of comorbidities in survival differences, we focus on cancers, which are the second leading cause of death and exhibit high levels of heterogeneity. We apply the GMM clustering framework to demonstrate their survival differences (Fig. S7).

### Distinct progression trajectory patterns among cluster groups

**Fig. 7:**
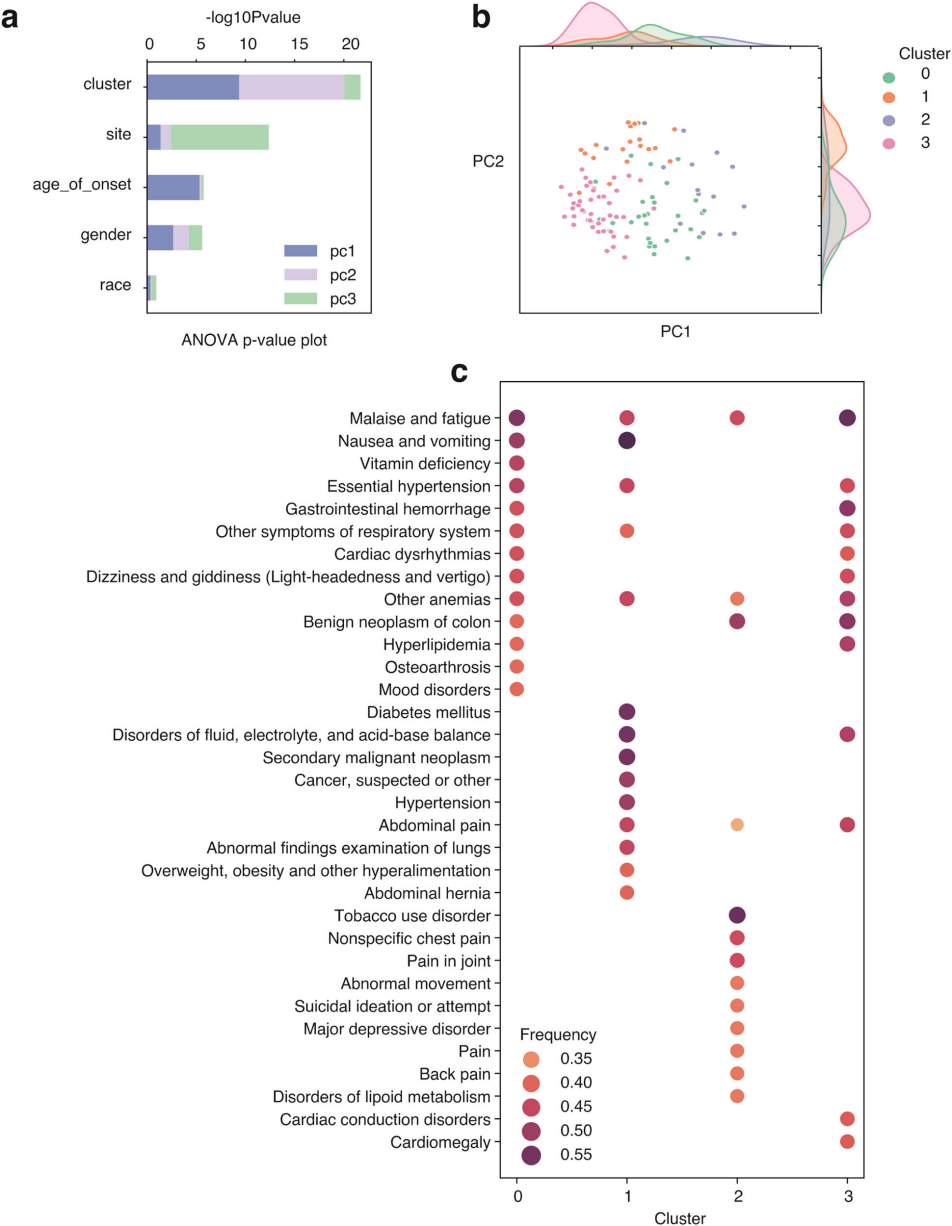
Longitudinal analysis revealed progression differences within each cluster group in CRC. **a** Barplot showing ANOVA results of negative log_10_ *P*-value of each variable in the *y*-axis, explaining variances of PCs in different colors. **b** Scatterplot showing the cluster groups in different colors is driven by PC1 and PC2. **c** Dot plot indicating phenotype (comorbidity) gain (*y*-axis) within each cluster after CRC onset. Color scales are used to indicate the fraction within each cluster obtaining a new phenotype corresponding to the *y*-axis.

We further evaluated the potential of using longitudinal embedding vectors to study the progression of diseases. We used CRC (phecode = 153) as an example, including all available patients with 10-year longitudinal data after a diagnosis of CRC (*n* = 110). We first performed Principal Components Analysis (PCA) on the longitudinal vectors, trying to decompose the changes in disease progression and analyze the variance. We included the first three PCs which explained 75.6% of the variance (Fig. 7a). Using analysis of variance (ANOVA), including the age of onset, race, gender, site, and clusters as covariates, we found that PC1 and PC2 are explained majorly by the cluster groups we identified using GMM (see session), then the age of onset, gender, sites, and races (Fig. 7a, b). While PC3 is explained mainly by sites (Fig. 7b). This result suggests that the variation in disease progression longitudinally is also captured by the clusters, indicating that individual cluster groups also have a different disease progression track, meaning that the cluster groups we identified capture the disease trajectories. To understand the differences in progression, we then analyzed the emerging phenotypes following the onset of the disease, revealing substantial differences among cluster groups (Fig. 7C). Besides the consistent occurrences of “Malaise and fatigue” and “Other anemias” across all four clusters, a few phenotypes were also present in three out of four clusters. These included “Essential hypertension,” “Gastrointestinal hemorrhage,” “Other symptoms of the respiratory system,” “Benign neoplasm of the colon,” and “Abdominal pain.” The remaining emerging phenotypes displayed radical variations across all four clusters, indicating distinct progression trajectories and varied comorbidity patterns. Given the substantial disparities in the progression of the four clusters, we investigated their disease patterns before the onset of the CRC. Our investigation revealed their initial divergence, as illustrated in Fig. S8. However, these disparities are not particularly prevalent; the maximum frequency across all four clusters is merely 21% (Fig. S8). This frequency can subsequently escalate to as high as 55% after the onset of the disease (Fig. 7c). These findings indicate that disease progression is highly heterogeneous, yet they reveal noticeable patterns regarding comorbidities. This observation indicates varying disease risks and underscores the potential for personalized medicine approaches.

## Discussion

Mining information from the EHR has become a crucial topic for several meaningful downstream implementations, such as disease prediction, patient phenotyping, and personalized medicine^14,49^. In this work, we developed a novel patient embedding method using EHR data. We integrated a three-step model architecture to accomplish this complex task and demonstrated several downstream applications. We evaluated the model's performance both internally and externally. Although performance on the external UW EHR data declined, the metrics score (Table 4) remains satisfactory, experimentally demonstrating the robustness of our model. We noticed drastic differences in model performance across various lengths of patient vectors (Fig. S9). This variation is related to the attention mechanism. The attention mechanism is designed for pairwise translation, where a sentence usually consists of a proper number of words. Therefore, when a patient sequence sparsely contains very few codes, the model lacks a strong co-occurrence pattern to recover the original codes.

Several other studies have explored the potential of representation learning to characterize diseases using the EHR with various machine learning architectures^10,11,13,50,51^. Most of these studies use different metrics and disease annotations, making it challenging to compare the performances. However, we did notice a trend of more complicated model architectures and downstream applications within the development of this new area, indicating a promising future for EHR representation learning. Among them, our model used a novel vocabulary embedding strategy to represent the diagnosis and procedure codes in the onset frequency domain. Our model has fewer components—only diagnosis and procedure codes—yet maintains high computational efficiency while achieving vigorous performance. To our knowledge, we are the first group to utilize a complex source of EHR data across 12 sites to perform the representation learning, resulting in a comprehensive and more generalized patient representation model. Importantly, we appear to be the only group that externally examined model performance, validating the results and demonstrating reproducibility using an External EHR dataset.

We demonstrated that a simple linear combination of the embedded features can manage disease onset predictions and bulk phenotyping tasks. The disease onset prediction itself is not only an evaluation of the embedding but also shedding new light on an automatic prediction tool for the alert system in the EHR regarding patient risks of future diseases.

In disease comorbidity analysis, we applied cluster algorithms and detected distinct comorbidity patterns within CRC and SLE. Meanwhile, we reproduced similar cluster results and revealed distinctions in overall survival in the UW cohort. In the progression analysis, we demonstrate that each cluster is linked to distinct phenotype gains. This suggests that the groups within the cluster are progressively different, indicating that our model reflects continuous variation rather than capturing a static moment. Moreover, this unsupervised method is data-driven and thus not limited to existing knowledge for EHR-based risk prediction and can uncover new disease patterns and potentially explain the progression differences of highly heterogeneous diseases. Further analysis using UW EHR data identified overall mortality disparities among different cluster groups, paving the way for further investigation of health outcome variations.

Together, these results suggest that partitioning patients into different subgroups can reveal discrepancies in disease progression and critical health outcomes. Although more efforts are needed to understand the complex comorbidity relationship, implementing an intelligent clinical decision support system to facilitate personalized medicine is feasible, leveraging the abundant patient data within the EHR. For instance, when the system notices that an HIV patient has recently been diagnosed with CRC, which is an extremely high risk, an early warning can inform the severity of the co-occurrence of these two phenotypes to facilitate on-time treatment. Thus, this system can identify urgency and achieve early healthcare to increase life expectancy. Besides actions in clinical application, identifying comorbidity patterns within a phenotype might indicate pathological, etiological, behavioral, or environmental similarities between co-occurring phenotypes. These comorbidity patterns can facilitate fundamental scientific advances in identifying molecular signatures of diseases and thus help us better understand the mechanisms.

Our model has a few notable limitations. First, our model only included diagnosis and procedure codes as embedding building blocks, lacking medications, labs, observations, and clinical notes due to the limitation of data sources. Without these variables, our model might lose certain meaningful information and limit the downstream analysis on medications, labs, etc. Besides, we used phecodes as surrogate phenotypes. Though phecodes have demonstrated their efficiency in large-scale EHR-based genetic studies, they lack granularity and may not be an appropriate use for some complicated phenotypes, such as depression^52^. Moreover, misdiagnosis is common in the EHR, which can cause potential issues that lead to deteriorated embedding qualities. A few studies have suggested diagnostic errors that impact timely healthcare, which in our case can lead to a misrepresentation of patients and a failure to capture meaningful patterns^53^^,54^. Finally, our patient data drawn from the eMERGE consortium might contain potential ascertainment bias during patient recruitment, meaning that there might be population structures that do not represent the general population of the United States. However, on the other hand, with only diagnosis and procedure codes available, our model still demonstrated great performance in several downstream analyses, such as bulk phenotyping, disease forecasting, comorbidity pattern study, and progression analysis. This is not surprising, as diagnoses and procedures are the most crucial information within the EHR for many downstream tasks. Although phecodes are new and still in development, there is evidence that phecodes can reproduce genetic findings and serve as a great proxy for phenotypes. To adjust for the potential biases caused by individual sites, we always included sites as covariates in our statistical analysis. Most importantly, we demonstrated the external validity of our model using the UW dataset and showcased the robustness of our performance with experimentally reproduced stable disease patterns, which were invariant across different cohorts.

In summary, our study developed a novel architecture for modeling patient learning using EHR data, highlighting the effectiveness of bulk phenotyping, disease forecasting, and variations in comorbidities. More importantly, we demonstrated that the disease trajectory differs within each cluster, providing insights for studying disease progression to understand its etiology and pathology. Future works may consider two directions among others. First, integrating additional data sources such as laboratory results, medications, and vital signs into the current model could enhance performance and enable more complex downstream analyses focused on health outcomes. Second, conducting detailed subgroup characterizations—such as applying topic modeling to patient data and analyzing complex clinical outcomes—could further refine our understanding of disease dynamics^25,55,56^.

## Methods

### Data

EHR data from the eMERGE Network (*n* = 102,740) were used to train and develop the patient embedding models. These included basic demographics (birth decade, gender, race, and ethnicity), patient diagnosis codes (ICD-10 and ICD-9), procedure codes (CPT-4), and age at diagnosis. We included a basic demographics summary of the eMERGE data in Supplementary Tables 1 and 2. The UW EHR data (*n* = 840,000) served as a validation set. Besides the same data elements mentioned above (birth decade, gender, race, ethnicity, diagnoses, and procedures), UW included overall mortality data, which is then used to evaluate the survival differences among clusters. A basic demographics summary of the UW data is in Supplementary Table 2.

### IRB

This study is approved by the IRB STUDY00015886: Generalizability Assessment of the eMERGE study on the University of Washington Medical Center Population. All data extracted from the University of Washington is under this IRB approval and stored on a HIPAA-compliant server.

### Autoencoder model structure

We collected all available codes, including ICD-9, ICD-10, and procedure codes. To ensure the quality of embedding, we kept all codes that appeared more than five times in the entire dataset, resulting in 34,851 unique codes. We used the week as a time interval unit and counted the number of appearances of each code across 0–4320 weeks, which equals 90 years. This produced a 34,851 × 4320-dimensional matrix, with each unique code represented by a vector of length 4320, and in the autoencoder model, this served as a feature for each code. The autoencoder model architecture is illustrated in Fig. 1a. The goal of this model is to condense the representation of the codes, producing a 34,851 × 50-dimensional matrix, where each code vector length is reduced from 4320 to 50. The non-linear functions in the autoencoder can identify complex patterns among the codes. In addition to using the traditional reconstruction error to train the model, we incorporated a cosine similarity loss function to maintain certain linear correlation properties among the codes. Moreover, the embedded layer contains a mean and standard deviation parameter, making this a variational autoencoder. We experimented with several different hyperparameters and selected the model with three layers that demonstrated the best performance, particularly in reconstruction loss and KL loss, to ensure that sampling closely resembles the original data (Supplementary Table 3). We trained this model using Adam optimizer^57^ with default parameters of beta1 and beta2, learning rate 1e−7, and batch size of 64 (Fig. S1, left). During the training, 5% of the training data is split and used as a validation set to evaluate the loss. The model is trained for 100 epochs, reaching a steady reconstruction loss and similarity loss. The embedded 50-dimensional codes serve as vocabularies in the transformer model to construct patient vectors.

### Transformer models structure

We utilize the transformer model, incorporating an attention mechanism to create patient vectors^26,27^. Our model employs a standard transformer model architecture (Fig. S1, middle), except that we did not include the positional embeddings for each code. As diagnoses do not always indicate the exact time of disease onset. It only denotes the starting point of potential treatment and systemic awareness of the presence of diseases, as many chronic diseases indeed happen way earlier than a hospital diagnosis. Thus, all events documented in the hospital within a specific year do not suggest the absolute sequential relationship in real life. Therefore, we aggregated all patient codes occurring within a year sequentially into a single vector, without requiring specific positional encoding for the codes within that year. The distribution of codes and patient longitudinal data is included in Supplementary Figure 2. After testing several setups with various hyperparameters, we selected *L* = 6, *H* = 10, dff = 2048, and *d* = 50 as they yielded the best results (Supplementary Table 4). The pre-trained embeddings of codes from the autoencoder serve as vocabularies. In the preprocessing step, we bin each patient’s codes by year, and for each year, we create a vector of codes to represent the temporal patient vector. The maximum vector length is set to be 250, considering that in one year, most patients should receive <250 codes from the hospital. For all 102,740 available patients, only 0.25% have more than 250 codes in a year, which is rare and likely only occurs in extremely severe cases. For patients having codes <250, we apply zero padding to the sequence and mark it as a special padding token. In total, there are 1,046,649 patient vectors for 102,740 patients. The goal of this transformer model is to use the current year’s codes to predict the future year’s codes, therefore, learning the relationship and progression longitudinally. Thus, we fit longitudinal patient event codes at year *i* as input, and the patient events at year *i* + 1 as output. During the training process, we masked 20% of the codes in each vector and let the model reconstruct the full sequence of vectors. We trained this model with Adam optimizer with beta1 = 0.9, beta2 = 0.98, epsilon = 1e−9, and a scheduled learning rate that gradually decreased to 0.002 at step 10,000. The batch size is set to 32, reducing the computation memory load. Again, 5% of the data is split and used as validation to evaluate the loss during the training.

### Sentence model structure and training detail

Inspired by the sentence embedding method^58^, we build a similar model architecture that takes two patients as input and gives two binary outputs. The first binary output indicates whether the two vectors of patients belong to the same patient (*is_same_patient* task). The second one assesses if one vector is the subsequent event of another vector (*is_next_event* task). We first use the global average pool of the embedding layer from the transformer model to represent patients as numerical vectors (Fig. S1, right). We then added a feed-forward structure of a pre-embedding layer and a 50-dimensional embedding layer to compute the complex interactions of the embedded sequence (Fig. S1, right). We use two different loss functions to optimize the two tasks mentioned above. The goal of the *is_same_patient* task is to minimize the mean square distance between two 50-dimensional embedded vectors, as an evaluation of the vector distance in geometric space. While the *is_next_event* task is optimized by a 2-layer feed-forward neural network constructed by concatenating the embedding of two vectors, this approach facilitates the learning of complicated relationships between two vectors. We only used two layers of feed-forward structure to avoid overfitting and to ensure it learned meaningful functions according to the theory that two layers of neural networks can simulate any form of continuous function^59^. We also found that there is limited performance improvement (only seen in *is_next_event* tasks) when having more layers (Supplementary Table 5). During the training, we randomly formed pairs of patient vectors as input and trained until saturation (no loss decrease). We found the model performance peaked at 5000 steps during training with a learning rate of 3e−4.

### Mapping of diagnosis code to phecodes

The mapping between ICD9 and ICD10 diagnosis codes to phecodes is done by referencing the PheWAS website https://phewascatalog.org/ through the phecodes mapping panel, counting at least one presence of a diagnosis code as a qualifying phenotype.

### Converting patient-year embeddings into patient embeddings

The patient-yearly embeddings (or longitudinal embeddings) are concatenated into patient embeddings by simply taking the average. This approach represents the sum of all patients’ yearly embedding vectors into a single vector that represents the current patient status, with normalization by the number of steps (the total number of vectors). For example, for a patient *k*, we sum all the year-embeddings from *t* = 1 to *t* = *n*, then we divide the sum by the number of steps *n* to get the mean vector, which is the patient embedding vector.

1 $${\rm{M}}{\rm{e}}{\rm{a}}{\rm{n}}\,{{\rm{v}}{\rm{e}}{\rm{c}}{\rm{t}}{\rm{o}}{\rm{r}}}_{k}=\frac{{\sum }_{t=1,\ldots n}{\rm{p}}{\rm{a}}{\rm{t}}{\rm{i}}{\rm{e}}{\rm{n}}{\rm{t}}\,{{\rm{v}}{\rm{e}}{\rm{c}}{\rm{t}}{\rm{o}}{\rm{r}}}_{k}^{t}}{n}$$

### Disease onset prediction and bulk phenotyping

Both disease onset prediction and bulk phenotyping are classification tasks in this work. In the disease onset prediction, for each phenotype, we collected all longitudinal vectors of patients and split them into before the onset versus after the onset group. The “before onset” longitudinal vectors comprise all patient longitudinal embeddings from one year before the onset age of a given phenotype. The “after onset” group includes patient longitudinal embeddings from the year of onset and subsequent years for that phenotype. We then used the longitudinal vectors to build a logistic regression classifier to perform classification tasks for the two groups of vectors. For example, for each disease *i*, for *j* in 1…50, *x*_*j*_ represents numerical features drawn from the embedding (embedding size of 50), the logistic regression prediction whether the disease *i* is already presented in the longitudinal vector or not:

2 $${{\rm{o}}{\rm{n}}{\rm{s}}{\rm{e}}{\rm{t}}}_{i}=\frac{1}{1+{e}^{\Sigma -{\beta }_{j}{x}_{j}}}$$

Bulk phenotyping is less complicated. For each phenotype (phenotype_*i*_), we computed the mean of vectors (meanVector_*k*_) across all time point (*t* within individual patients (patient_*k*_), resulting in a single vector for each patient (as oftentimes referenced as patient embedding).

Logistic regression models were then applied to these vectors to discern whether patients exhibit a specific phenotype or not, as defined by phecodes.

3 $${{\rm{p}}{\rm{h}}{\rm{e}}{\rm{n}}{\rm{o}}{\rm{t}}{\rm{y}}{\rm{p}}{\rm{e}}}_{i}=\frac{1}{1+{e}^{\Sigma -{\beta }_{j}{x}_{j}}}$$

Regarding both disease onset prediction and bulk-phenotyping, for each phenotype, we used 80% of the sample for training and evaluated the model performance on the 20% unseen data.

### Comorbidity cluster analysis

We performed comorbidity analysis within a single phenotype to reflect the heterogeneity. The Gaussian mixture model is used to first group samples into clusters. We chose the number of clusters based on the Bayesian information criteria (BIC). Then, within individual clusters, we performed logistic regression for each comorbidity (defined by phecodes), including the cluster (using one versus the rest), age of onset, sites, gender, ethnicity, and race as covariates to predict the comorbidity. e.g.

4 $${{\rm{p}}{\rm{h}}{\rm{e}}{\rm{n}}{\rm{o}}{\rm{t}}{\rm{y}}{\rm{p}}{\rm{e}}}_{i}=\frac{1}{1+{e}^{{\beta }_{0}+{\beta }_{1}{\rm{a}}{\rm{g}}{\rm{e}}+{\beta }_{2}{\rm{s}}{\rm{e}}{\rm{x}}+{\beta }_{3}{\rm{s}}{\rm{i}}{\rm{t}}{\rm{e}}{\rm{s}}+{\beta }_{4}{\rm{g}}{\rm{e}}{\rm{n}}{\rm{d}}{\rm{e}}{\rm{r}}+{\beta }_{5}{\rm{e}}{\rm{t}}{\rm{h}}{\rm{n}}{\rm{i}}{\rm{c}}{\rm{i}}{\rm{t}}{\rm{y}}+{\beta }_{6}{\rm{c}}{\rm{l}}{\rm{u}}{\rm{s}}{\rm{t}}{\rm{e}}{\rm{r}}}}$$

Adjusting for multiple tests (*n* = 1855 comorbidities), we used Benjamini–Hochberg adjusted *p*-values < 2e−5 as the significance level.

### Model evaluation

We evaluated the external performance of the model on both the eMERGE dataset (internal) and the UW EHR data (external). As described in the model architecture session, there are 1,046,649 patient vectors for 102,740 patients for the eMERGE dataset. Externally, we pulled available de-identified UW EHR data from the year 2000 to 2020, including *n* = 840,000 patients. We evaluated the transformer model and the patient-vector model output. For eMERGE data, we randomly selected 32,000 events (1000 steps with batch size 32) for the evaluation task. For UW data, the evaluation of the transformer model utilized randomly drawn 5000 patients, consisting of *n* = 66,776 events in total. The goal was to investigate the ability of the model to reconstruct the original codes given the patient vector. Performance for the eMERGE dataset is included in the “Results” section and Table 1. The performance for the UW dataset is shown in Table 3. The S-BERT model evaluation used 500 randomly drawn patients in 5 iterations (100 randomly drawn patients in each iteration), consisting of 34,633 and 29,587 events for eMERGE (Table 2) and UW datasets (Table 4), respectively. Note that the evaluation process is based on the number of events, not the number of patients. Thus, though the number of patients is relatively small compared to the total sample size, the number of events already reflects consistent and robust performance. Moreover, the summary statistics of the evaluation metrics demonstrated extremely low variance, suggesting robustness against large numbers of patients.

### Benchmarking disease onset prediction

We performed benchmarking against two previous models, Deep Patient and BEHRT. To our knowledge, Deep Patient is one of the initial patient embeddings in the early field that achieved greater performance than the traditional matrix decomposition method in future disease prediction. BEHRT is the state-of-the-art model built upon the BERT model in predicting future diseases^30^. We trained the Deep Patient model and the BEHRT model based on the proposed model architecture and hyperparameters reported in their original paper until they converged. We benchmarked our model (PatientEmbedding) and the transformer model (PatientEmbedding model without S-BERT fine-tuning) against these other models. To compare embedding quality and benchmark results, we extracted embeddings from each model and applied logistic regression (as described in the “Disease onset prediction” section) to predict future disease onset. For BEHRT, a contextual model with six embedding layers, we used the last layer, as it is commonly regarded as the most effective layer for downstream tasks. All models were trained on the eMERGE dataset and evaluated on an unseen dataset (UW EHR), ensuring robust performance validation. The detailed results are shown in Supplementary Table 6. Among the four models, BEHRT achieved the best performance. Our transformer model (without fine-tuning with S-BERT) achieved close results, with 0.022 lower median AUROC and 0.012 lower mean AUROC. Although BEHRT achieved a slightly better performance than the PatientEmbedding model, we reason that the PatientEmbedding model after S-BERT fine-tuning is optimal for downstream tasks, such as heterogeneity analysis. The outstanding and stable performance on heterogeneity analysis could be due to the tuning of the PatientEmbedding model.

### Longitudinal comorbidity analysis

For each patient, the longitudinal vectors after the disease onset are collected. We used 10 years as the endpoint of disease progression, meaning that we collected 10 longitudinal vectors for each available patient (*n* = 110), using CRC as an example. We again computed the average of the vectors within individual patients and used them to perform the PCA (Fig. 7b). We defined the occurrence of phenotypes as phenotypes that only occur after the onset of diseases, which did not exist before the disease onset. The new phenotypes were first counted and then normalized by the number of patients within each cluster, represented as frequency. We then selected the top 15 phenotypes within each cluster and represented them in the plot (Fig. 7c).

### Software versions and code availability

The patient embedding model is implemented through tensorflow version 2.3.0, and the high-level API tensorflow.keras, version 2.4.0. Models were trained using an NVIDIA 2060-Super GPU with 8 GB RAM. The code for running the model and synthetic data is available at the GitHub repo https://github.com/suxian06/language-model-based-patient-embedding/tree/main. Data analysis using TSNE, PCA, GMM, and BIC was implemented through the scikit-learn version package 0.24.2 in Python. Logistic regression and analysis of variance (ANOVA) were implemented using statsmodels, version 0.12.2. Plots were generated using Matplotlib version 3.4.3, Seaborn 0.11.2. The online interactive charts were generated using Altair version 5.0.1. Survival analyses (Kaplan–Meier plot and Log-rank test) were performed using the scikit-survival packages in Python, version 0.14.0. Log-rank test used in differentiating the subgroup survival was also based on the scikit-survival package (compare_survival function). Standardization, numerical operations, and data cleaning are done with numpy version 1.23.0 and scipy version 1.6.2.

## Supplementary information

Supplementary Figures

## Data Availability

The data used for this study are available upon request at the eMERGE Network.

## Code availability

Code to reproduce the results is posted on the GitHub page: https://github.com/suxian06/language-model-based-patient-embedding/tree/main.
