# Author Correction: Transformer patient embedding using electronic health records enables patient stratification and progression analysis

**DOI:** 10.1038/s41746-026-02678-3

**Published:** 2026-06-11

**Authors:** Su Xian, Monika E. Grabowska, Iftikhar J. Kullo, Yuan Luo, Jordan W. Smoller, Theresa L. Walunas, Wei-Qi Wei, Gail Jarvik, Sean Mooney, David Crosslin

**Affiliations:** 1 Department of Biomedical Informatics and Medical Education, University of Washington, Seattle, WA USA; 2 Department of Biomedical Informatics, Vanderbilt University Medical Center, Nashville, TN USA; 3 Department of Cardiovascular Medicine and the Gonda Vascular Center, Mayo Clinic Rochester Minnesota, Rochester, MN USA; 4 Department of Preventive Medicine, Northwestern University Feinberg School of Medicine, Chicago, IL USA; 5 Psychiatric and Neurodevelopmental Genetics Unit, Center for Genomic Medicine, Massachusetts General Hospital, Boston, MA USA; 6 Center for Precision Psychiatry, Department of Psychiatry, Massachusetts General Hospital, Boston, MA USA; 7 Department of Medicine, Northwestern University Feinberg School of Medicine, Chicago, IL USA; 8 Department of Medicine, Division of Medical Genetics, University of Washington, Seattle, WA USA; 9 Department of Genome Sciences, University of Washington, Seattle, WA USA; 10 Center for Information Technology, National Institutes of Health, Bethesda, MD USA; 11 Department of Medicine, Division of Biomedical Informatics and Genomics, Tulane University, New Orleans, LA USA

**Keywords:** Gastrointestinal cancer, Tumour heterogeneity, Cancer, Diseases, Cancer, Immunological disorders

Correction to: *npj Digital Medicine* 10.1038/s41746-025-01872-z, published online 14 August 2025

“In this article the funding information ‘Research reported in this publication was partly supported by the National Institute of General Medical Sciences of the National Institutes of Health under Award Number P20GM152305. The content is solely the responsibility of the authors and does not necessarily represent the official views of the National Institutes of Health.’ was omitted. The original article has been corrected.”

