# Supplementary material for: Transformer patient embedding using electronic health records enables patient stratification and progression analysis
Source: NPJ Digit Med. 2025 Aug 14;8:521. doi: 10.1038/s41746-025-01872-z (PMC12354887; doi:10.1038/s41746-025-01872-z)
Supplement: Supplementary file 1 — Supplementary Figures [file 41746_2025_1872_MOESM1_ESM.docx]

# Supplementary Figure

Supplementary Figure 1. Illustration of the model architecture.

From left to right, the figure shows how we started from an autoencoder (left) that generates embeddings for the vocabularies to the transformer model (middle) for patient yearly embedding. Finally, S-BERT concatenates and fine-tunes the transformer encoder, yielding patient embeddings.


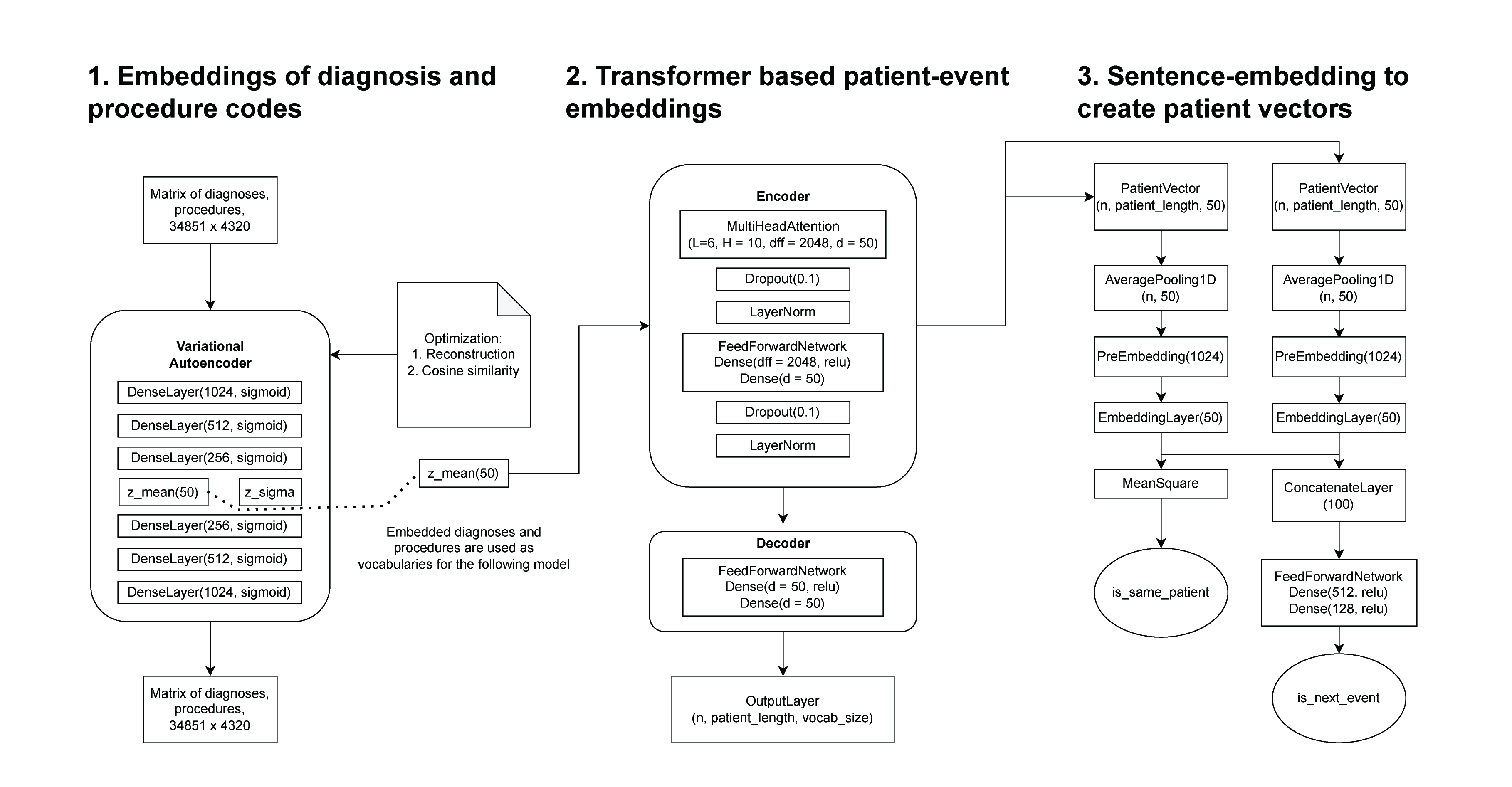


Supplementary Figure 2. Distributions of the eMERGE data and UW data.


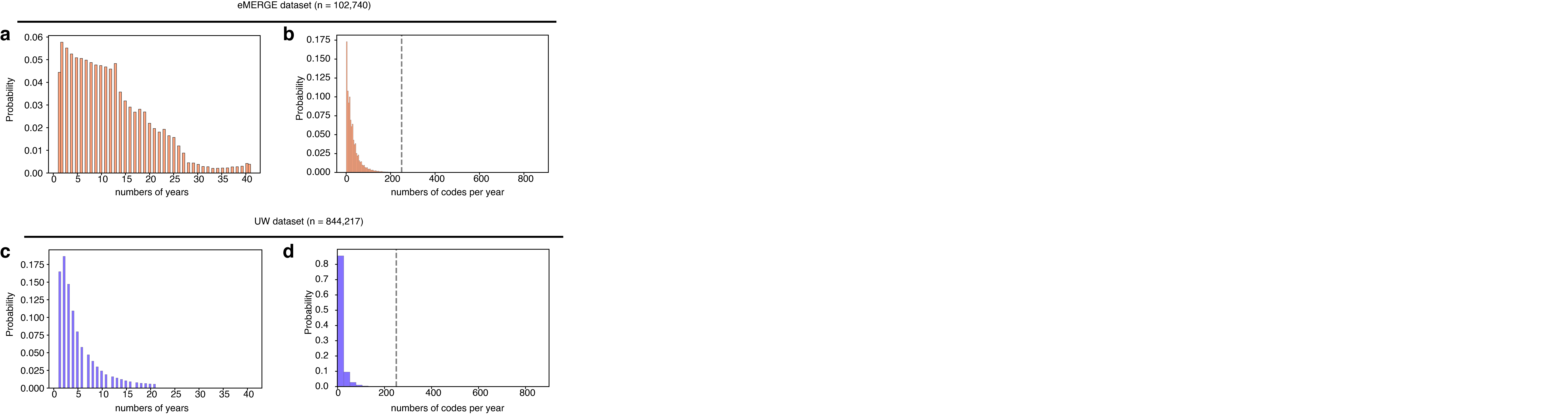


a, c. Distribution of the years of longitudinal patient data in eMERGE dataset (a) and UW dataset (c).

b, d. Distribution of the numbers of codes in each yearly longitudinal patient vector after binning all visits within a year for eMERGE dataset (b) and UW dataset (d).

Supplementary Figure 3. (a) Disease onset prediction illustration and (b) disease versus non-disease state prediction.


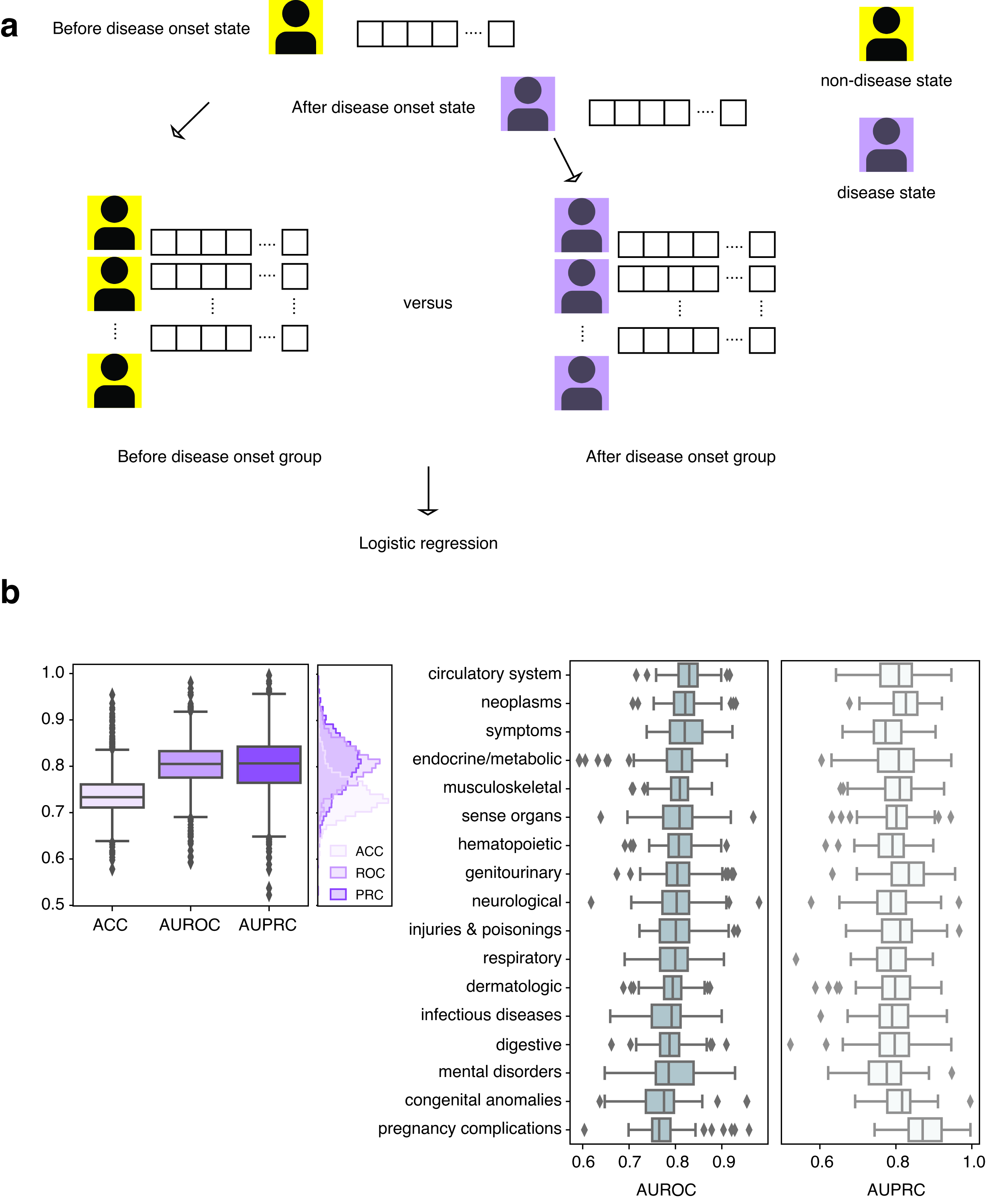


1. Illustration of predicting disease versus non-disease status using patient vectors.
2. Performances of disease versus non-disease state prediction. Boxplot on the left side showing accuracy (ACC), AUROC, and AUPRC distribution for all 1,855 phenotypes. Boxplot on the right showing AUROC and AUPRC grouped by different disease classes.

Supplementary Figure 4. Illustration of onset prediction and bulk phenotyping.


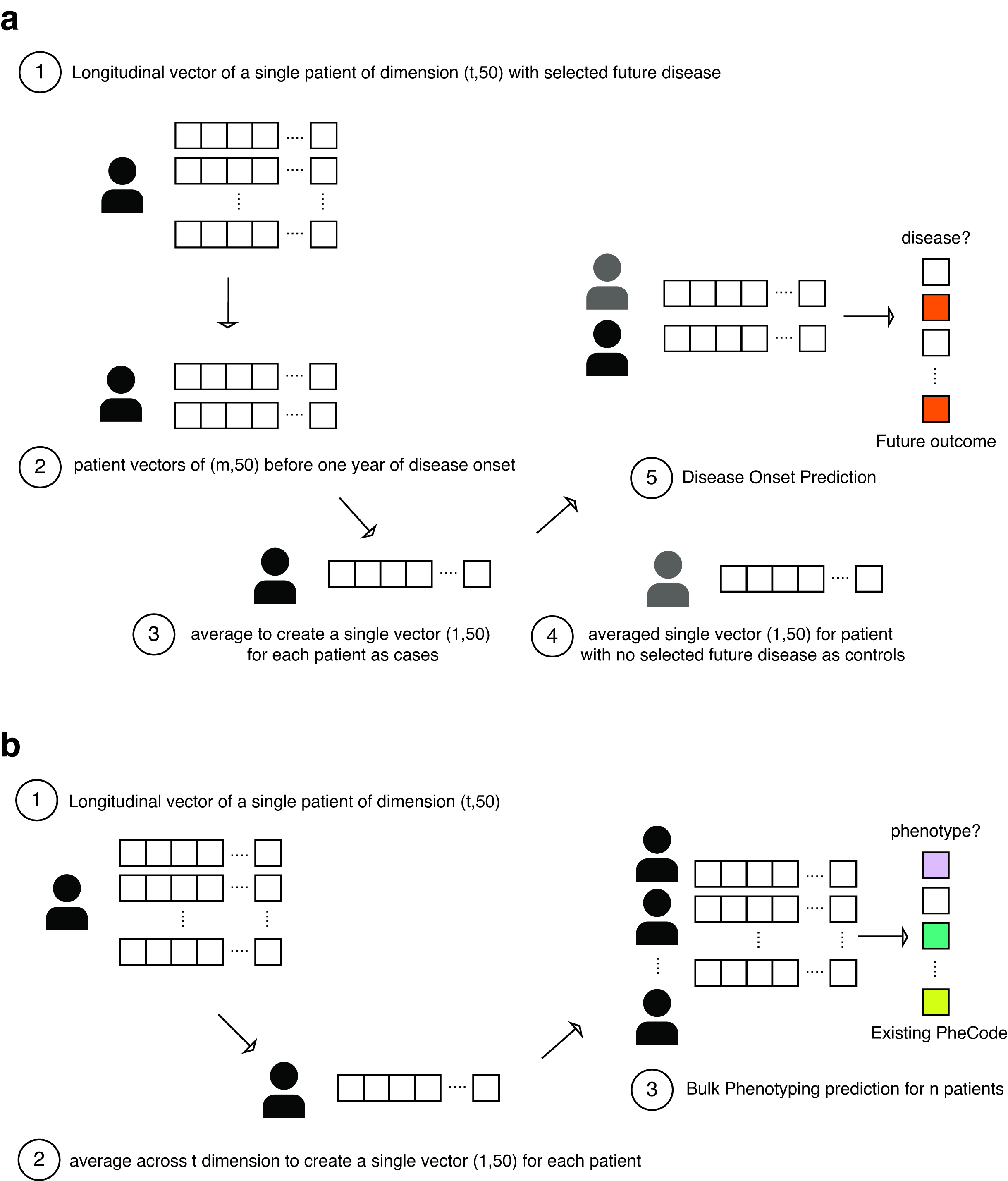


1. Illustration each step of onset prediction tasks.
2. Illustration each step of bulk phenotyping tasks.

Supplementary Figure 5. Bayesian Information Criteria (BIC) curve for model selection. (a, b) for eMERGE cohort and (c, d) for the UW cohort.


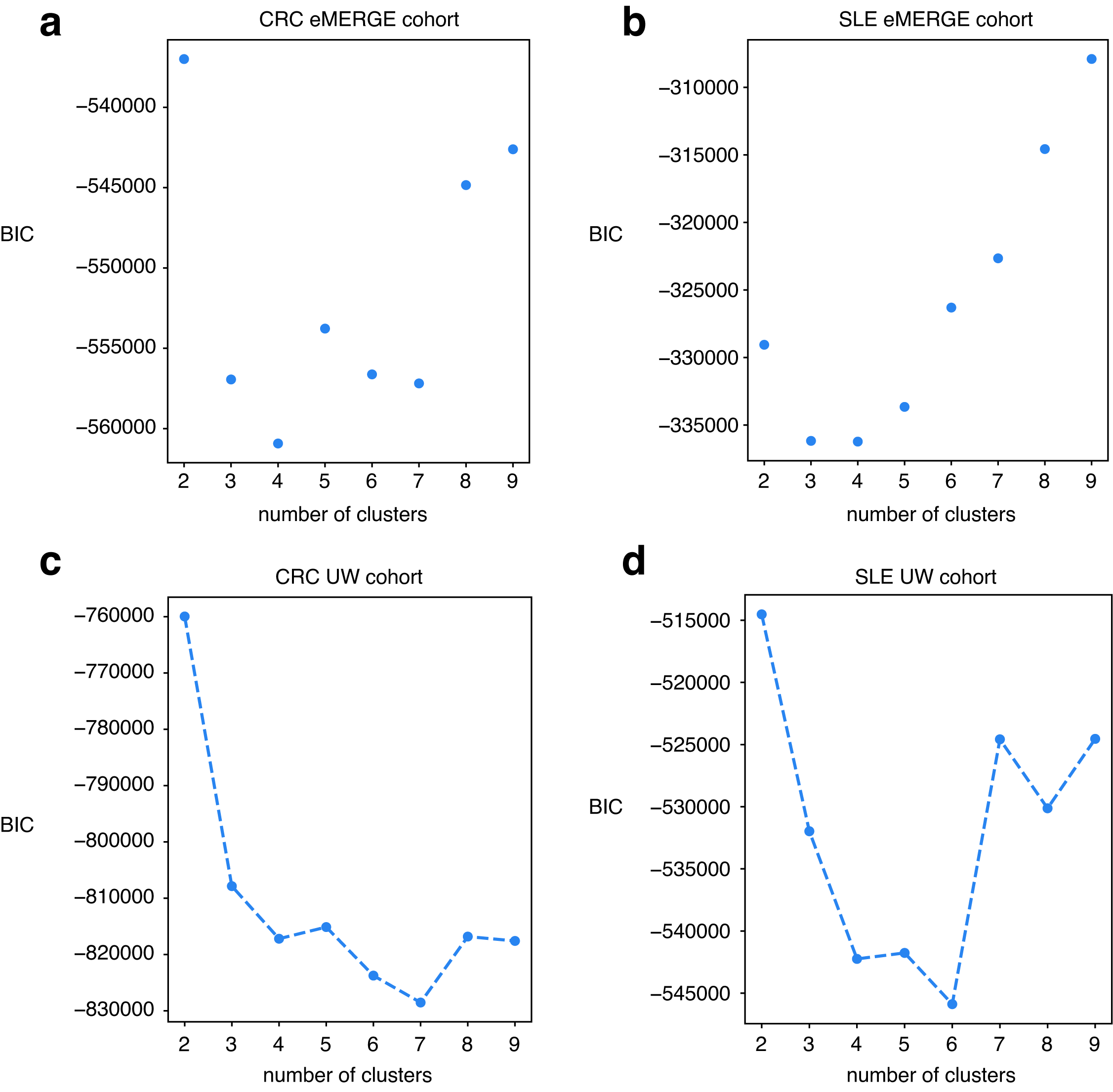


Supplementary Figure 6. Model performance on (a, b) disease onset prediction and (c, d) bulk phenotyping using the external UW cohort.

Performances on (a, b) disease onset prediction and (c, d) bulk phenotyping. The left side of each panel (a, c) shows the box plot representing AUROC distribution categorized by disease class according to phecodes and the right side (b, d) shows the relationship between sample size (positive/negative ratio) and AUPRC.


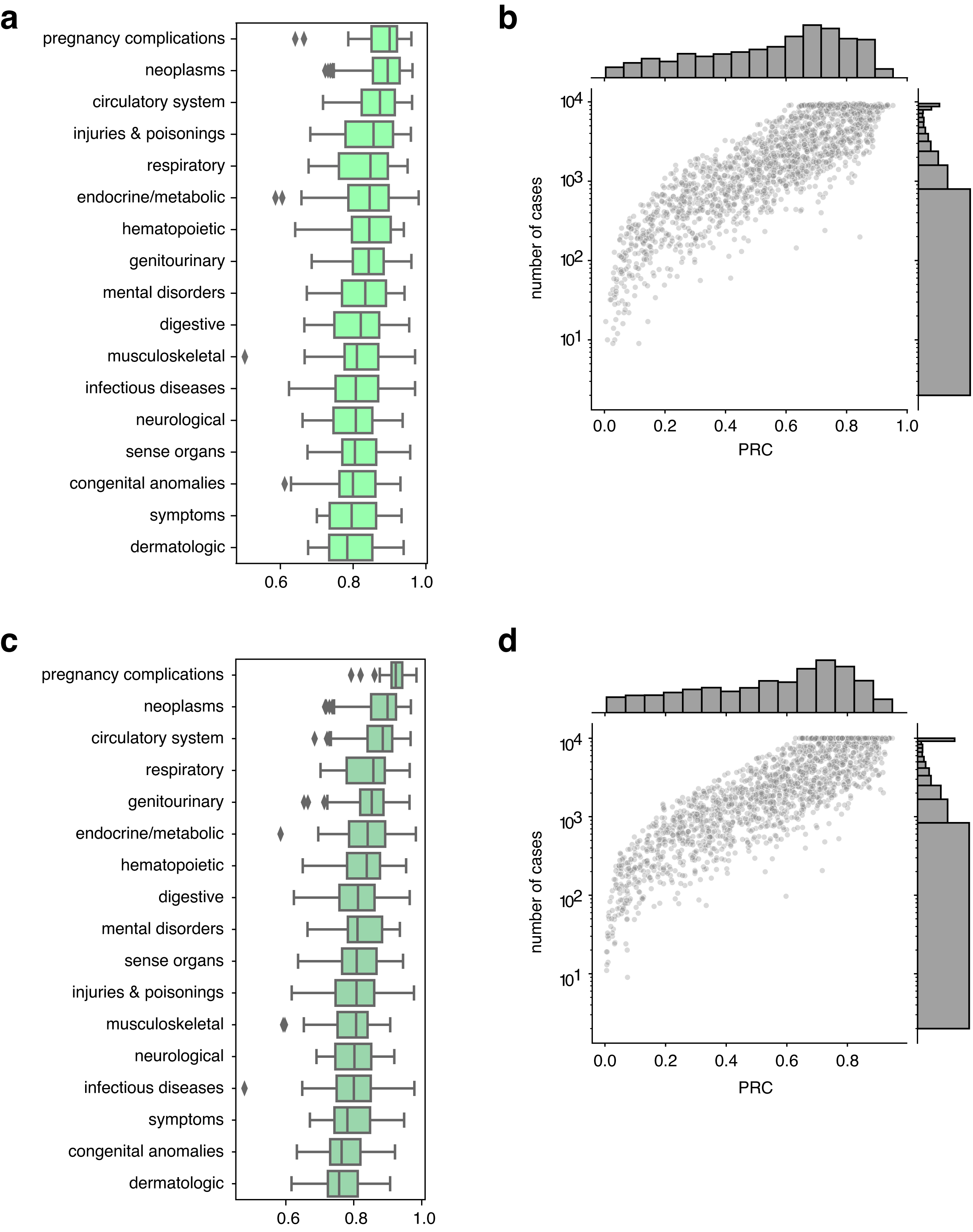


Supplementary Figure 7. Survival curve of multiple cancer types in the UW cohorts based on suggested clustering results revealing drastic differences in survival times.

Kaplan-Meier curves reveal differences in 10-year overall survival within each cluster group of multiple cancer types defined by phecodes.


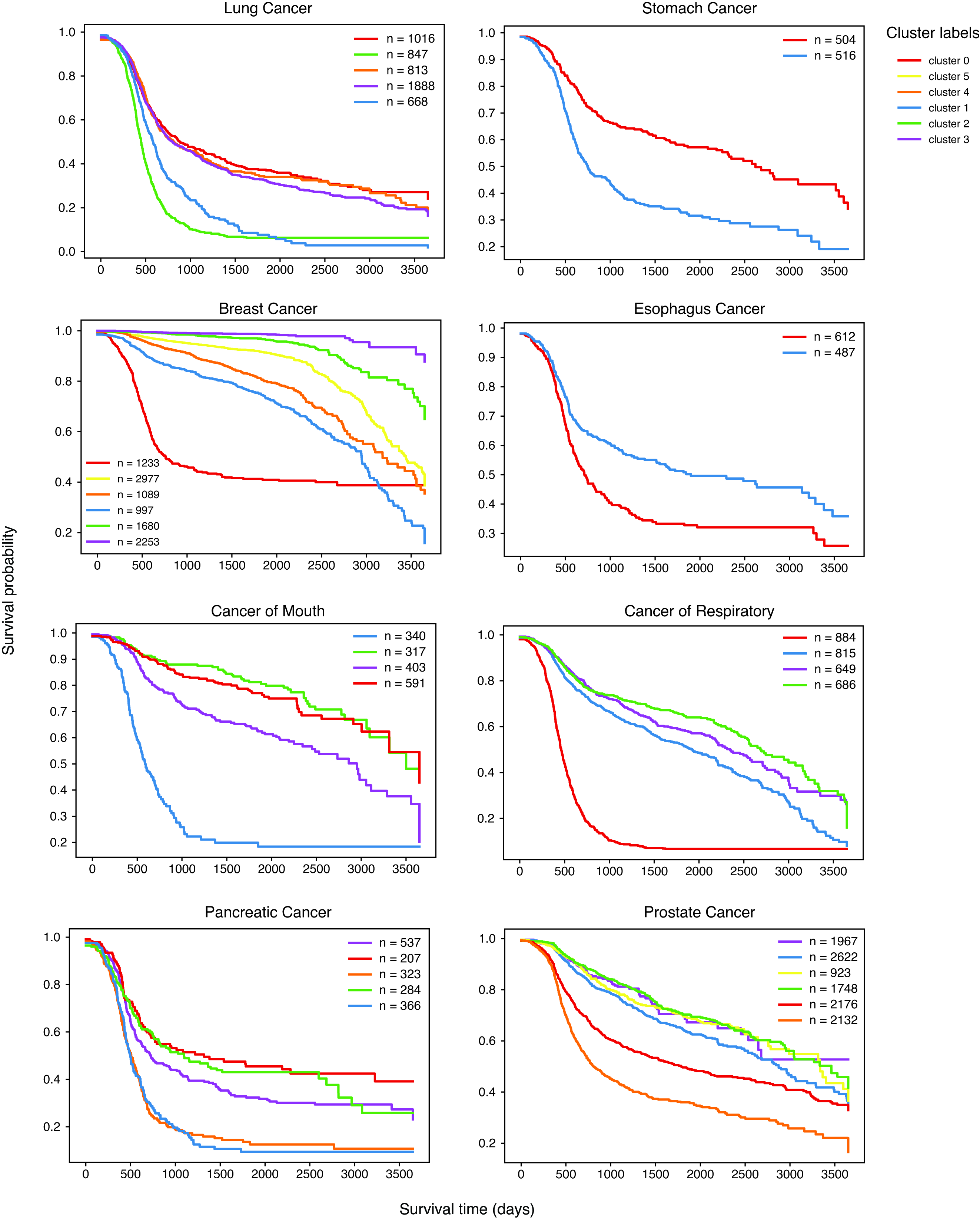


Supplementary Figure 8. Comorbidity differences within each cluster group in CRC before onset of CRC.

Dot plot indicating phenotype (comorbidity, y-axis) within each cluster before CRC onset. Color scales are used to indicate the fraction within each cluster that has a phenotype corresponding to the y-axis.


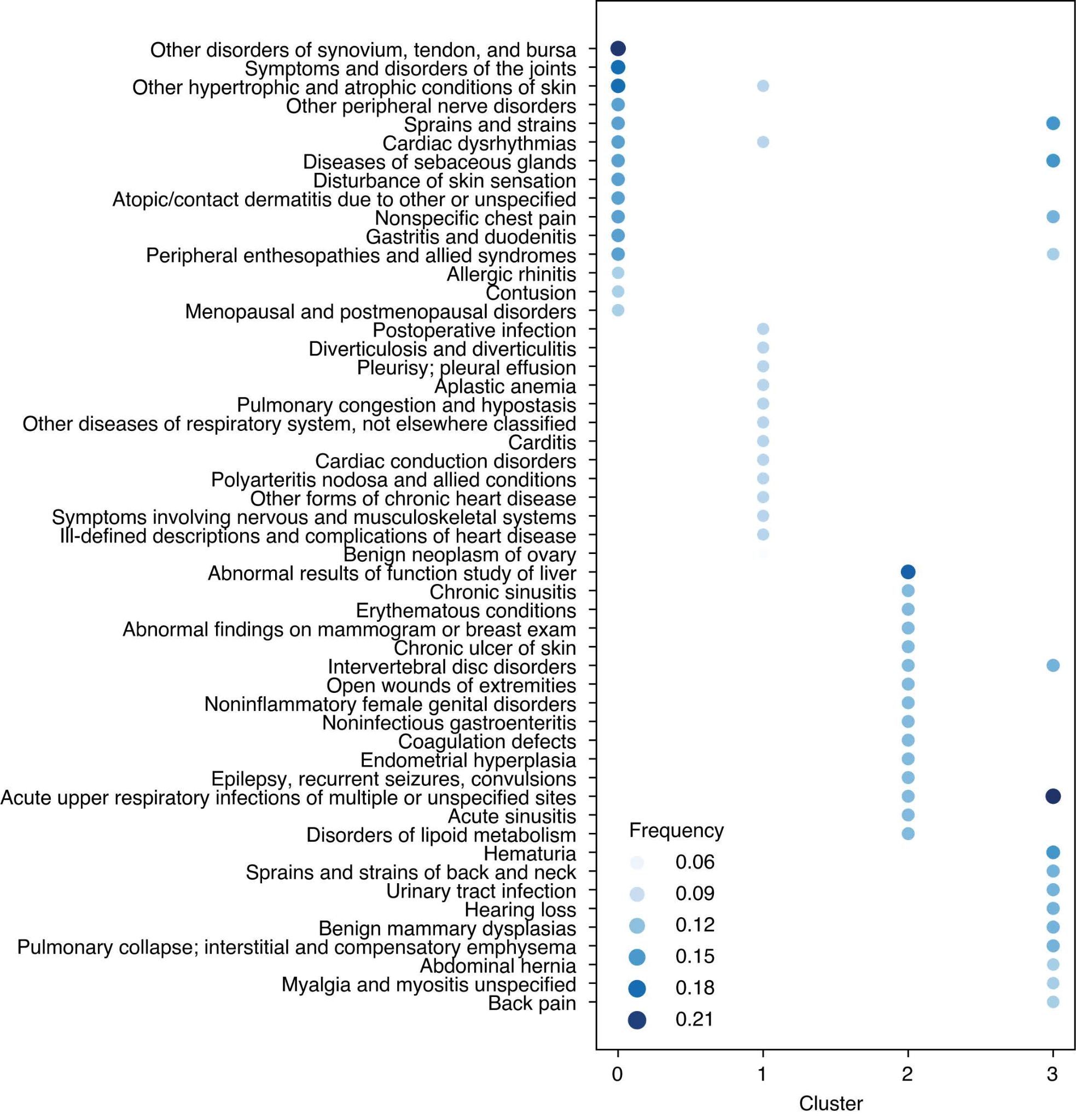


Supplementary Figure 9. Performance (precision and recall) differences in patient events with different numbers of codes.


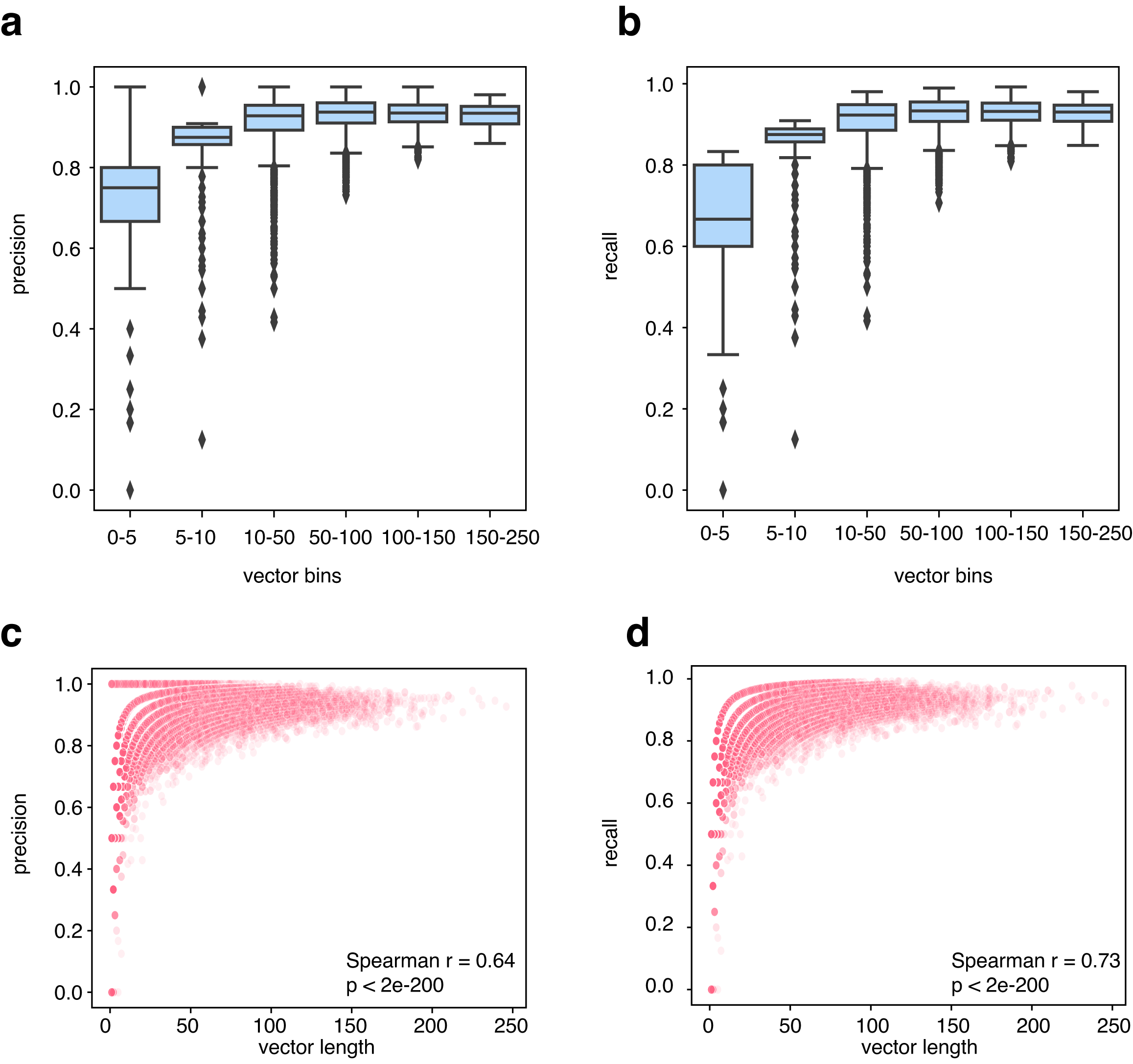


a - b. Precision (a) and recall (b) based on the numbers of codes binned into ranges in each patient event (x-axis).

c - d. Same relational plot of a - b but in continuous manner without binning numbers of codes into ranges.

# Supplementary Tables

Supplementary Table 1. Demographics of the eMERGE dataset, represented by sites.

| Sites | N samples | Age (mean + std) | Sex (female) |
| --- | --- | --- | --- |
| Harvard | 29.62% (29,697) | 63.98±17.24 | 16,339 (55.02%) |
| Vanderbilt | 20.82% (20,878) | 71.41±17.05 | 11,480 (54.99%) |
| Children’s Hospital of Philadelphia | 10.42% (10,448) | 25.23±5.67 | 4,823 (46.16%) |
| Mayo Clinic | 9.03% (9,059) | 80.06±13.49 | 4,365 (48.18%) |
| Mount Sinai | 6.20% (6,217) | 68.99±15.06 | 3,680 (59.19%) |
| Cincinnati Children’s Hospital | 5.85% (5,863) | 21.76±7.18 | 2,436 (41.55%) |
| Northwestern | 4.70% (4,712) | 67.07±14.98 | 3,898 (82.72%) |
| Marshfield | 4.62% (4,634) | 84.92±9.66 | 2,802 (60.47%) |
| Geisinger | 2.95% (2,958) | 77.04±15.92 | 1,418 (47.90%) |
| Kaiser Permanente/UW | 2.90% (2,904) | 91.31±7.26 | 1,641 (56.51%) |
| Columbia | 1.98% (1,986) | 68.39±20.03 | 967 (48.69%) |
| Boston Children’s Hospital | 0.91% (916) | 22.09±5.10 | 373 (40.72%) |

Supplementary Table 2. Demographics breakdowns of the eMERGE (training dataset) and UW dataset (external evaluation data).

| Sites | N samples | Age (mean + std) | Sex (female) | Race |
| --- | --- | --- | --- | --- |
| eMERGE | 102,740 | 62.78±24.02 | 54,211(52.77%) | White, 76,581 (74.53%)  Black or African American, 15,476 (15.06%)  Unknown, 9,362 (9.11%)  Asian, 1,186 (1.15%)  American Indian or Alaska Native, 118 (0.11%)  Native Hawaiian or Other Pacific Islander, 17 (0.02%) |
| University of Washington | 844,217 | 51.59±22.69 | 451,909(53.53%) | White, 552,554 (65.45%)  Asian, 81,868 (9.70%)  Unavailable or Unknown, 49468 (5.86%)  Black or African American, 37,959 (4.50%)  Declined to Answer, 25,979 (3.24%)  Black or African-American, 20,759 (2.46%)  American Indian or Alaska Native, 7,100 (0.84%)  Native Hawaiian or Other Pacific Islander      4,491 (0.53%)  American Indian or Native Alaskan              2,921 (0.35%)  Native Hawaiian or Pacific Islander            1,969 (0.23%)  Mixture (More than one)  59,149 (7.01%) |

Supplementary Table 3. Hyperparameter searching for the autoencoder model.

The best performance is ***emphasized*** and with asterisk (*) indicate the model choice for this work.

| Model | n_Layer | d | Construction loss | KL loss | Similarity loss |
| --- | --- | --- | --- | --- | --- |
| 1 | 2 | 50 | 58117.9 | 1.12 | ***122.6*** |
| ***2**** | 3 | 50 | 58114.7 | ***0.98*** | 211.1 |
| 3 | 4 | 50 | 58018.5 | 8.13 | 1803.9 |
| 4 | 3 | 100 | 58031.8 | 3.49 | 1589.5 |
| 5 | 4 | 100 | ***57503.7*** | 51.38 | 3253.0 |

Supplementary Table 4. Hyperparameter searching for the transformer model.

The best performance is ***emphasized*** and with asterisk (*) indicate the model choice for this work.

| Model | n_Head | n_Layer | Dff (dimension of feed-forward NN) | d_model (embedding size) | Loss (best model) |
| --- | --- | --- | --- | --- | --- |
| 1 | 10 | 2 | 1024 | 50 | 1.6999 |
| 2 | 10 | 2 | 2048 | 50 | 1.7294 |
| 3 | 25 | 2 | 1024 | 50 | 1.6976 |
| 4 | 25 | 2 | 2048 | 50 | 1.7194 |
| ***5**** | 10 | 6 | 2048 | 50 | ***1.5968*** |
| 6 | 25 | 6 | 2048 | 50 | 1.6245 |

Supplementary Table 5. Hyperparameter searching for SBERT model.

The best performance is ***emphasized*** and with asterisk (*) indicate the model choice for this work.

| Model | n_Layer | Loss (best model) | Is_same_patient accuracy | Is_following_event accuracy |
| --- | --- | --- | --- | --- |
| ***1**** | 2 | ***0.5497*** | ***0.9524*** | 0.7857 |
| 2 | 3 | 0.5984 | 0.9412 | 0.8529 |
| 3 | 4 | 0.6938 | 0.8108 | ***0.9189*** |

Supplementary Table 6. Benchmarking results against Deep Patient and BEHRT

| Model | Median AUROC | Mean AUROC | N parameters |
| --- | --- | --- | --- |
| Deep Patient | 0.744 (0.677,0.829) | 0.747±0.089 | 21,132,110 |
| PatientEmbedding (after SBERT) | 0.837 (0.770,0.893) | 0.830±0.074 | 10,323,019 |
| Transformer (without SBERT) | 0.869 (0.841,0.894) | 0.866±0.036 | 10,102,040 |
| BEHRT | 0.891 (0.851,0.920) | 0.878±0.060 | 12,661,296 |
